# Supplementary material for: Hit-and-run epigenetic editing prevents senescence entry in primary breast cells from healthy donors
Source: Nat Commun. 2017 Nov 13;8:1450. doi: 10.1038/s41467-017-01078-2 (PMC5684409; doi:10.1038/s41467-017-01078-2)
Supplement: Supplementary file 1 — Supplementary Information [file 41467_2017_1078_MOESM1_ESM.pdf]

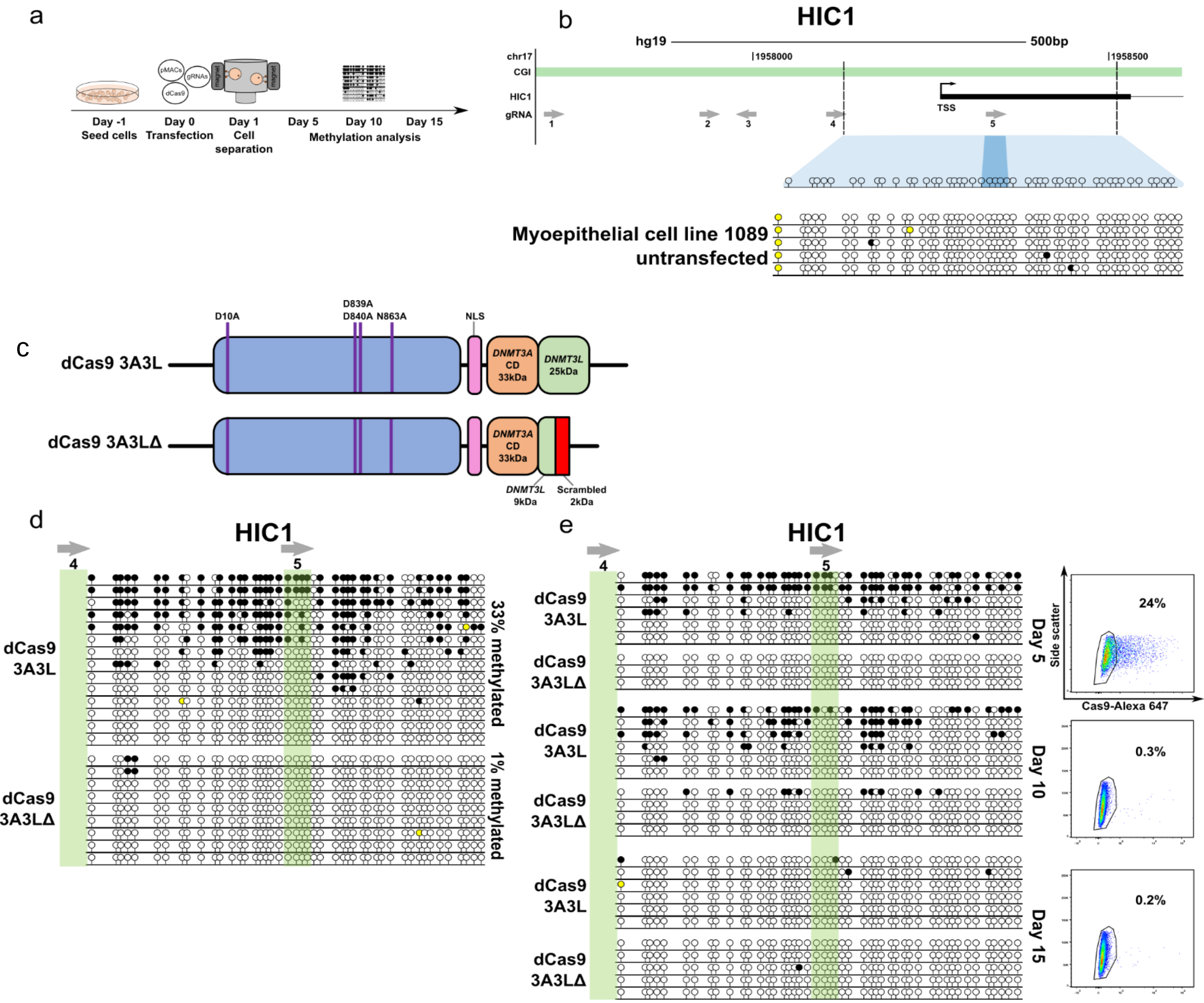

Supplementary Figure 1. Establishing transfection method with epigenetic editing constructs in 1089 cells. (a) Schematic depicting experimental protocol, transfected cells were enriched using magnetic sorting via the MACSelect system. At day 5, 1089 cells were split and reseeded or used for methylation analysis by bisulfite cloning. The methylation at *HIC1* was further analysed at 10 and 15 days after transfection. (b) A schematic depicting the gRNA location (grey arrows) in relation to the CGI (in green) and the TSS in the promoter of *HIC1*; the arrows point towards the PAM sequence. The dotted vertical lines represent the region analysed by bisulfite cloning and below are the locations of individual CpGs scaled according to their genomic distribution. The dark blue shading depicts which CpGs are overlapped by gRNA #5. Bisulfite cloning data below shows the methylation status of this region in untransfected 1089 cells. (c) The Cas9 sequence is mutated resulting in the following amino acid substitutions: D10A, D839A, D840A and N863A; rendering Cas9 catalytically inactive. dCas9 3A3L contains the catalytic domain of mouse *Dnmt3a* (amino acids 624-908, approximately 33kDa) and *Dnmt3l* (amino acids 208-421, approximately 25kDa). The dCas9 3A3LΔ construct has a 100bp duplication of DNA sequence within *Dnmt3l*, which results in only the first 81 amino acids out of 225 being correctly assembled followed by 19 amino acids which are not present in *Dnmt3l* before an in frame stop codon prematurely terminates translation. NLS, nuclear localisation signal. (d) Bisulfite cloning analysis five days after 1089 cells were transfected with either dCas9 3A3L or 3A3LΔ and the five gRNAs directed to the *HIC1* CGI. Numbered grey arrows and green shading depict the location of the gRNAs in relation to CpGs. Each line represents a single clone, filled and empty lollipops represent a single methylated or unmethylated CpG, respectively. A similar increase was seen in three independent biological replicates. (e) DNA methylation analysis from the total 1089 cell population 5, 10 and 15 days after transfection with either dCas9 3A3L or 3A3LΔ. Corresponding flow cytometry analysis demonstrating the typical percentage of Cas9^{+ve} cells at those time points is shown in the plots to the right.

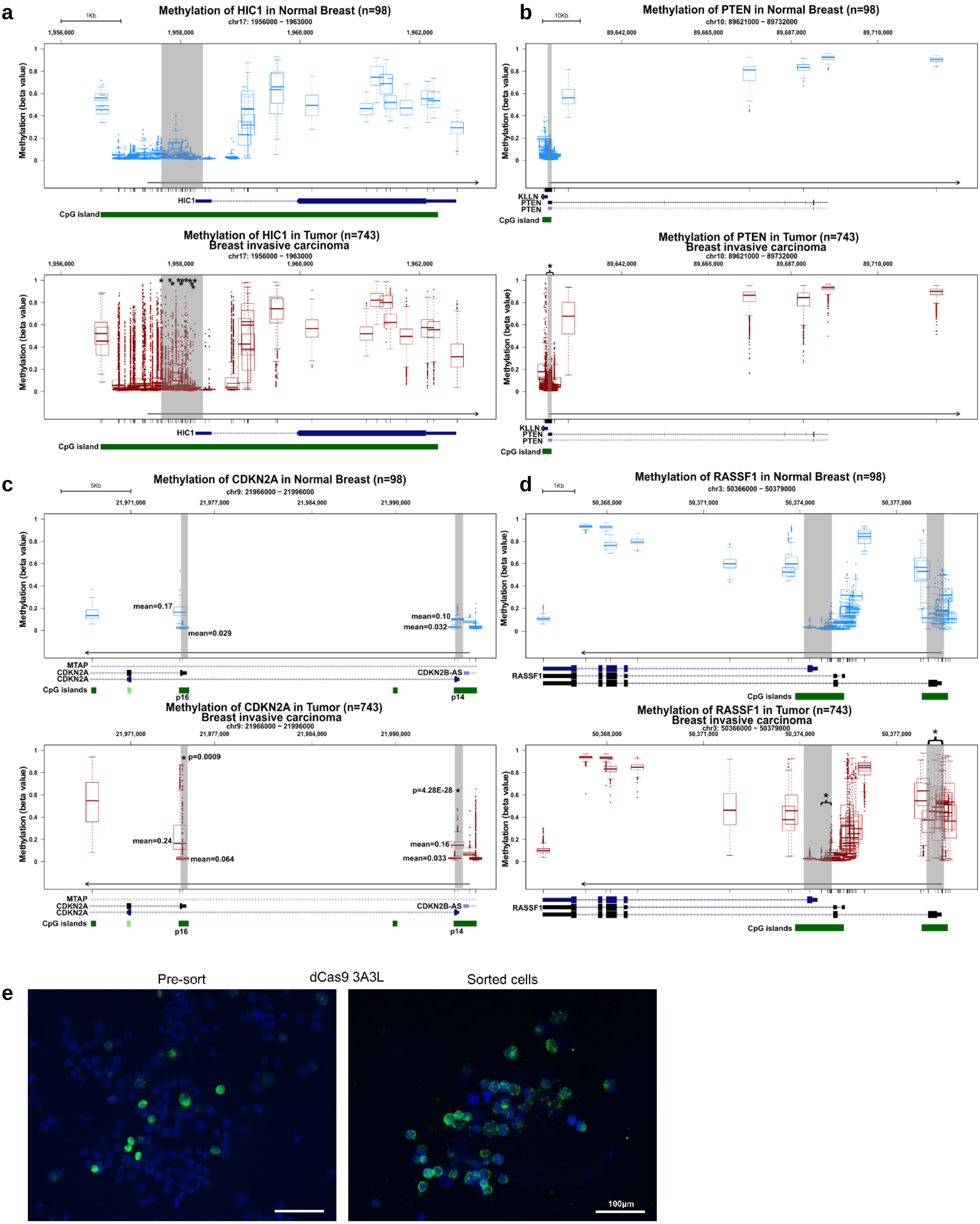
a
Methylation of HIC1 in Normal Breast (n=98)
chr17: 1956000 – 1963000
Methylation of HIC1 in Tumor (n=743)
Breast invasive carcinoma
Methylation (beta value)
HIC1
CpG island
b
Methylation of PTEN in Normal Breast (n=98)
chr10: 89621000 – 89732000
Methylation of PTEN in Tumor (n=743)
Breast invasive carcinoma
KLLN
PTEN
c
Methylation of CDKN2A in Normal Breast (n=98)
chr9: 21966000 – 21996000
mean=0.17
mean=0.029
mean=0.10
mean=0.032
Methylation of CDKN2A in Tumor (n=743)
Breast invasive carcinoma
p=0.0009
p=4.28E-28
mean=0.24
mean=0.064
mean=0.16
mean=0.033
MTAP
CDKN2A
CDKN2B-AS
CpG islands
p16
p14
d
Methylation of RASSF1 in Normal Breast (n=98)
chr3: 50366000 – 50379000
Methylation of RASSF1 in Tumor (n=743)
Breast invasive carcinoma
RASSF1
e
Pre-sort
dCas9 3A3L
Sorted cells
100μm

Supplementary Figure 2. Normal and tumour tissue methylation data of targeted tumour suppressors. dCas9 3A3L targeted regions are hypomethylated in primary breast tissue and become hypermethylated in some breast cancers. We performed data mining using the web tool Wanderer that utilises publically available Illumina HumanMethylation450 BeadChip methylation array data taken from The Cancer Genome Atlas (TCGA) to assess methylation levels at the promoters of (a) *HIC1*, (b) *PTEN*, (c) *CDKN2A* and (d) *RASSF1* in normal breast tissue and invasive carcinoma. Average DNA methylation from 98 normal samples and 743 invasive carcinoma samples is displayed as a box plot per probe. Schematic showing the localisation of the DNA methylation data in relation to CGI (green bar) and TSS are below each plot. Grey shaded region highlights the genomic area of interest. All available data is shown as a box plot per probe (mean ± standard deviation, normal n = 98, invasive breast carcinoma n = 748; Wilcoxon rank sum test, *, $p < 0.05$ for probes within the grey shaded area). (e) Immunofluorescence images of dCas9^{+ve} 1089 cell line (in green) 2 days after transfection with dCas9 3A3L and 10 gRNAs (targeting *HIC1* and *RASSF1*) before magnetic sorting (left panel) and after magnetic sorting using the pMACS receptor (right panel). Scale bar 100 µm, DAPI staining in blue.

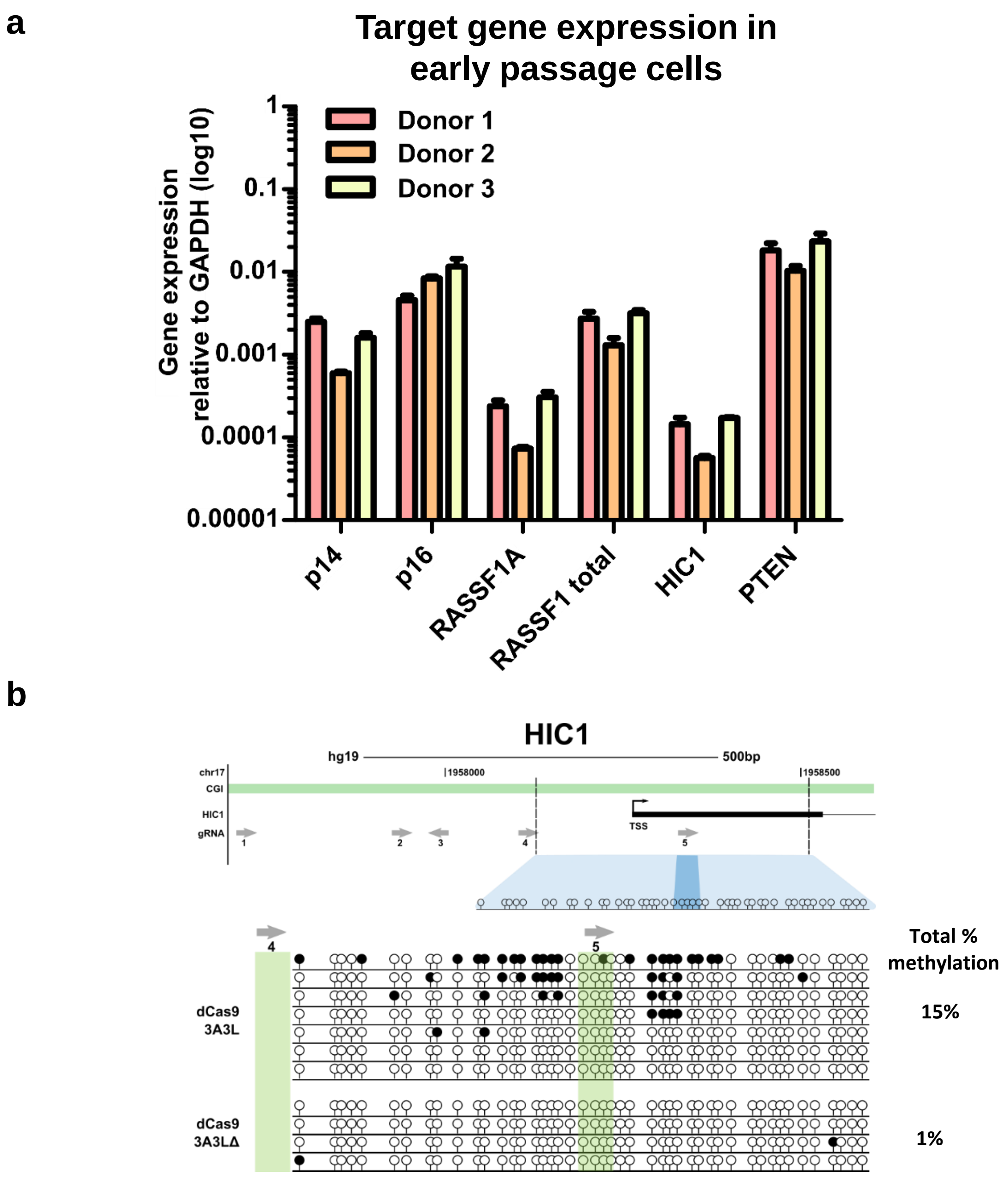

Supplementary Figure 3. Gene expression measured by qPCR of target genes (a) in early passage (passage 2) primary myoepithelial cells from donor 1 (red), donor 2 (orange) and donor 3 (yellow) after growing cells in culture for 7 days. Gene expression is relative to *ACTB* (mean ± SEM). (b) Schematic depicting the gRNA locations (grey arrows; pointing towards the PAM sequence), CGI (green bar) and TSS (black bar) of the *HIC1* gene. The dotted lines represent the region analysed by bisulfite cloning and below are the locations of individual CpGs scaled according to their genomic distribution. The dark blue shading depicts which CpGs are overlapped by gRNA #5. Lower panel shows bisulfite cloning results from the *HIC1* locus five days after transfecting primary myoepithelial cells from donor 1 with pMACs, 26 gRNAs (targeting *HIC1*, *RASSF1*, *PTEN* and *CDKN2A*) and dCas9 3A3L or 3A3LΔ as indicated (including magnetic sorting at day 2).

a

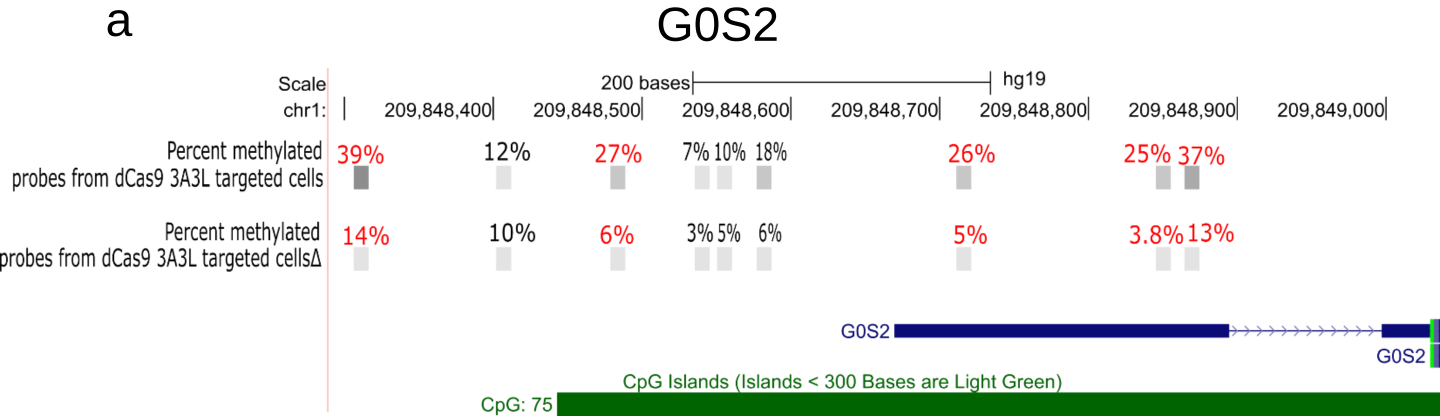

b

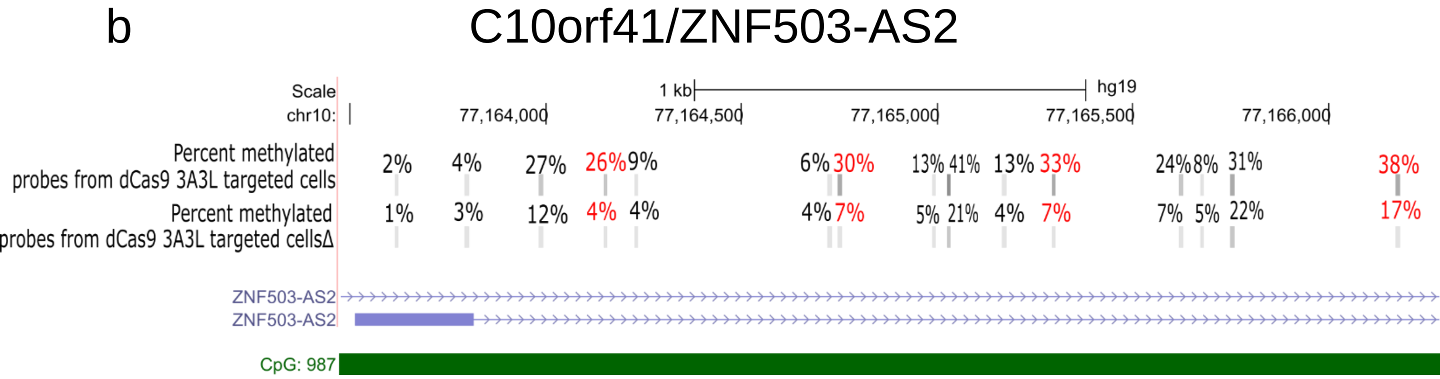

Supplementary Figure 4. EPIC array DNA methylation data of *G0S2* and *C10orf41/ZNF503-AS2*. Primary myoepithelial cells from donor 1 were transfected with pMACS, dCas9 3A3L or 3A3LΔ and 26 gRNAs (targeting *HIC1*, *RASSF1*, *PTEN* and *CDKN2A*), magnetically sorted at day 2, and harvested 10 days post-transfection. Each rectangle represents the location of a probe and the values show the percentage methylated in 3A3L (top track) or 3A3LΔ (bottom track) targeted cells. Values in red represent increases in methylation that were statistically significant and greater than 20% after analysis using the *ChAMP* (v. 2.8.3) package in R (n = 3; p > 0.01; Benjamini-Hochberg correction). The probes are shown in relation to the CGI (in green) and the TSS (in blue) of (a) *G0S2* and (b) *C10orf41/ZNF503-AS2*.

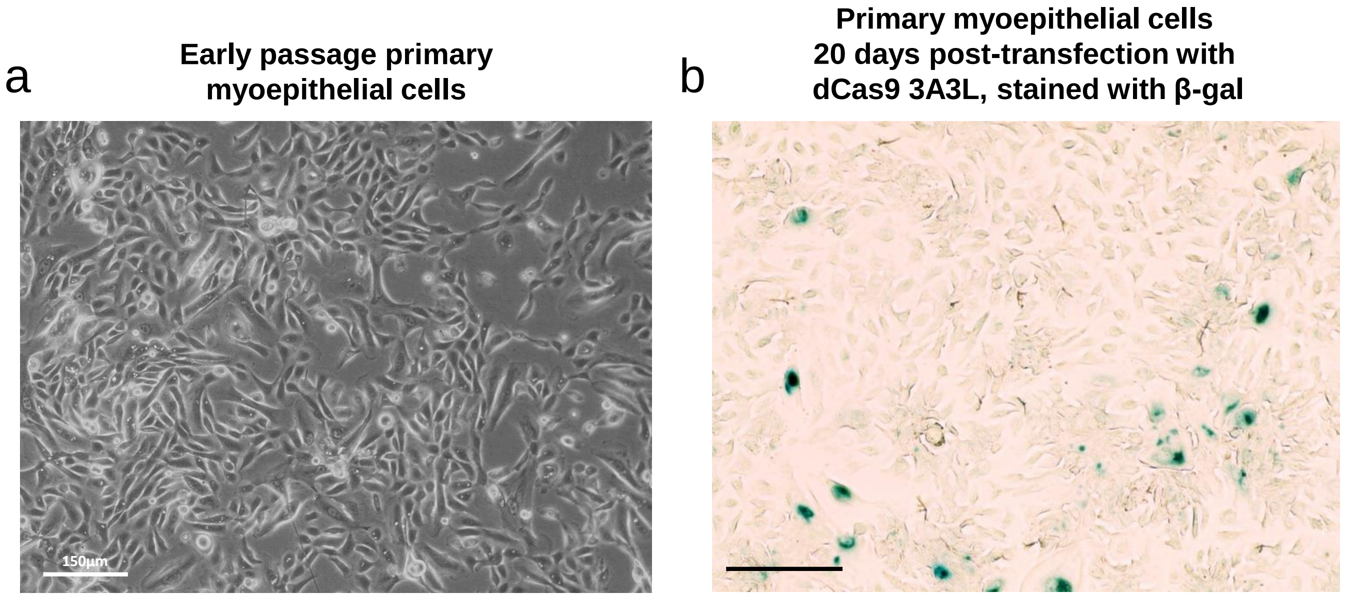

Supplementary Figure 5. Morphology of proliferating cells (a) Light microscopy image showing morphology of primary myoepithelial cells from donor 1, 3 days after seeding from frozen stock. Scale represents 150 µm. (b) Light microscopy image of primary myoepithelial cells from donor 1, 20 days after transfection with dCas9 3A3L and 26 gRNAs (targeting *HIC1*, *RASSF1*, *PTEN* and *CDKN2A*), then staining for presence of β-gal. Blue/green colouring depicts β-gal^{+ve} cells. Scale bar represents 125 µm.

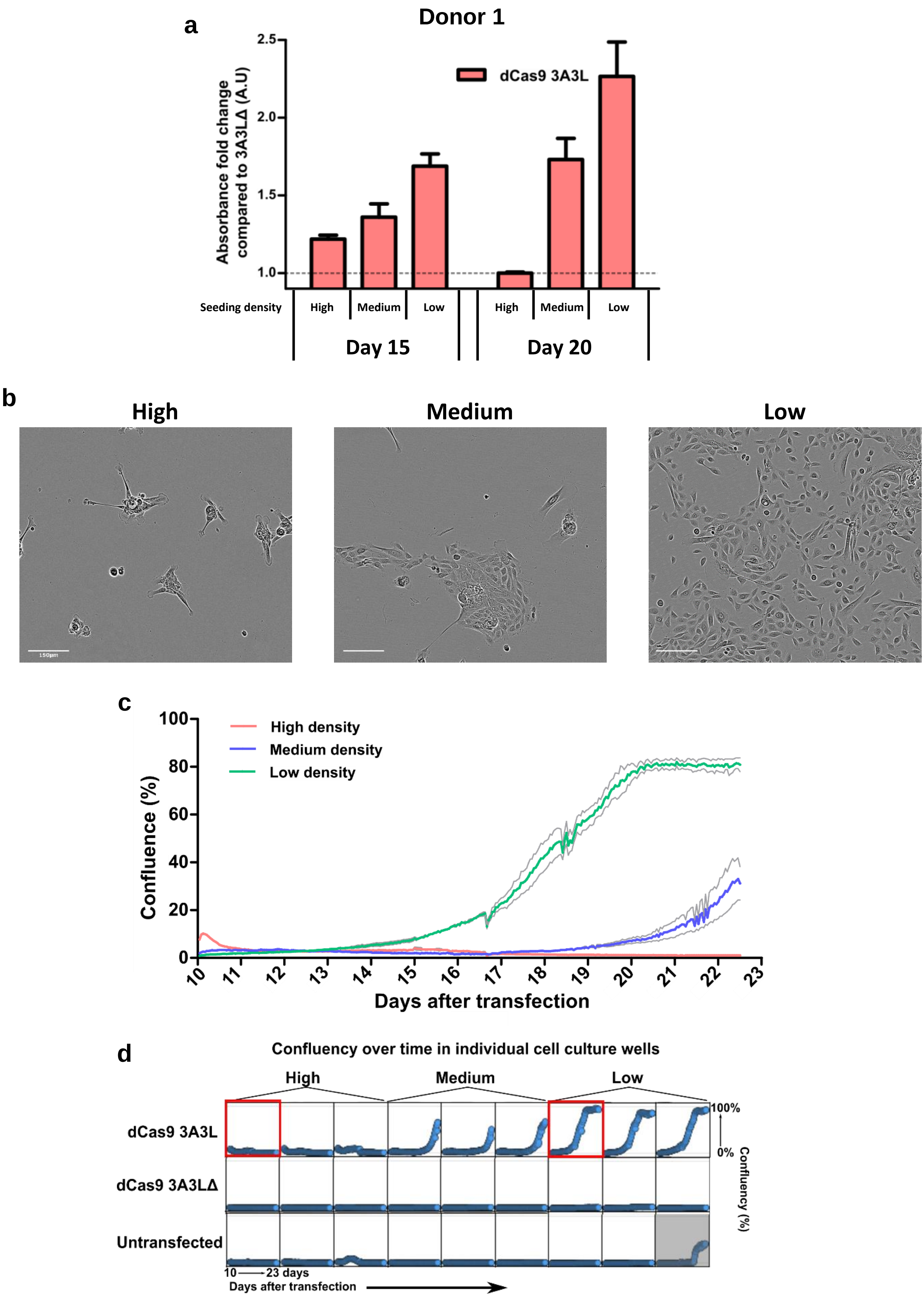

a
Donor 1
dCas9 3A3L
Absorbance fold change compared to 3A3LΔ (A.U)
Seeding density
High
Medium
Low
Day 15
Day 20
b
High
Medium
Low
c
High density
Medium density
Low density
Confluence (%)
Days after transfection
d
Confluency over time in individual cell culture wells
dCas9 3A3L
dCas9 3A3LΔ
Untransfected
100%
0%
Confluency (%)
10 → 23 days
Days after transfection

Supplementary Figure 6. Seeding density effect on proliferation in primary myoepithelial cells. (a) Primary myoepithelial cells from donor 1 were transfected with gRNAs (targeting *HIC1*, *RASSF1*, *PTEN* and *CDKN2A)*, dCas9 3A3L or 3A3LΔ and pMACs. Cells were magnetically sorted at 2 days post-transfection and at day 10 were reseeded into a 96 well plate at 3120 cells cm^{-1} (± 10%; high density), 1560 cells cm^{-1} (± 10%; medium density) or 780 cells cm^{-1} (± 10%; low density) and proliferation was measured via absorbance at days 15 and 20. The dotted line represents the average absorbance from three dCas9 3A3LΔ targeted wells at each timepoint. The graph shows the average difference in absorbance as fold change compared to 3A3LΔ average (mean ± SEM, n = 3). (b) Light microscopy images of cells at post-transfection day 20 that were seeded on day 10 at high, medium or low density; bar represents 150 µm. (c) Change in percentage confluency of cells reseeded at day 10 at high (pink), medium (blue) or low (green) density over time; confluency percentage was measured using images taken by the IncuCyte (1 image $hour^{-1}$ of three locations per well) by creating an image collection and processing definition using the IncuCyte ZOOM confluence processing software. Data are displayed as percentage confluency (mean ± SEM, n = 3). (d) Example replicates from IncuCyte data: each square represents a well from a 96 well plate, dCas9 3A3L (top row), dCas9 3A3LΔ (middle row) and untransfected (bottom row) donor 1 primary myoepithelial cells are shown. Cells were seeded at day 10 post-transfection at 3120 cells cm^{-1} (± 10%; high density), 1560 cells cm^{-1} (± 10%; medium density) or 780 cells cm^{-1} (± 10%; low density). Each square shows data depicting percentage confluency (y-axis) over time (x-axis), for a total for 13 days. Each data point is the average confluency from three individual images per well $hour^{-1}$. Wells with grey shading were excluded as they dried out over the course of the experiment. Red boxes show wells which were used to make movies of cell growth after seeding 3120 cells cm^{-1} (Supplementary Movie 1) and 780 cells cm^{-1} (Supplementary Movie 2).

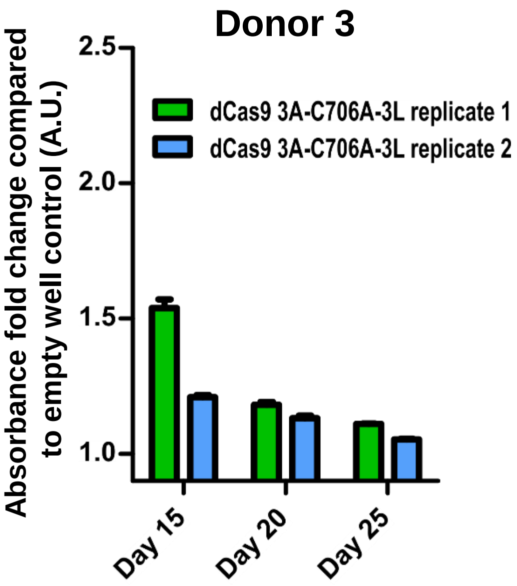

Supplementary Figure 7. Targeting dCas9 3A-C706A-3L does not result in senescence prevention. Proliferation was assessed using a colorimetric assay 15, 20 and 25 days after targeting the four genes in two replicate experiments with dCas9 3A-C706A-3L without magnetic sorting using donor 3 myoepithelial cells. The data is shown as fold change compared to the average absorbance from 3 wells without cells (mean ± SEM, n = 3).

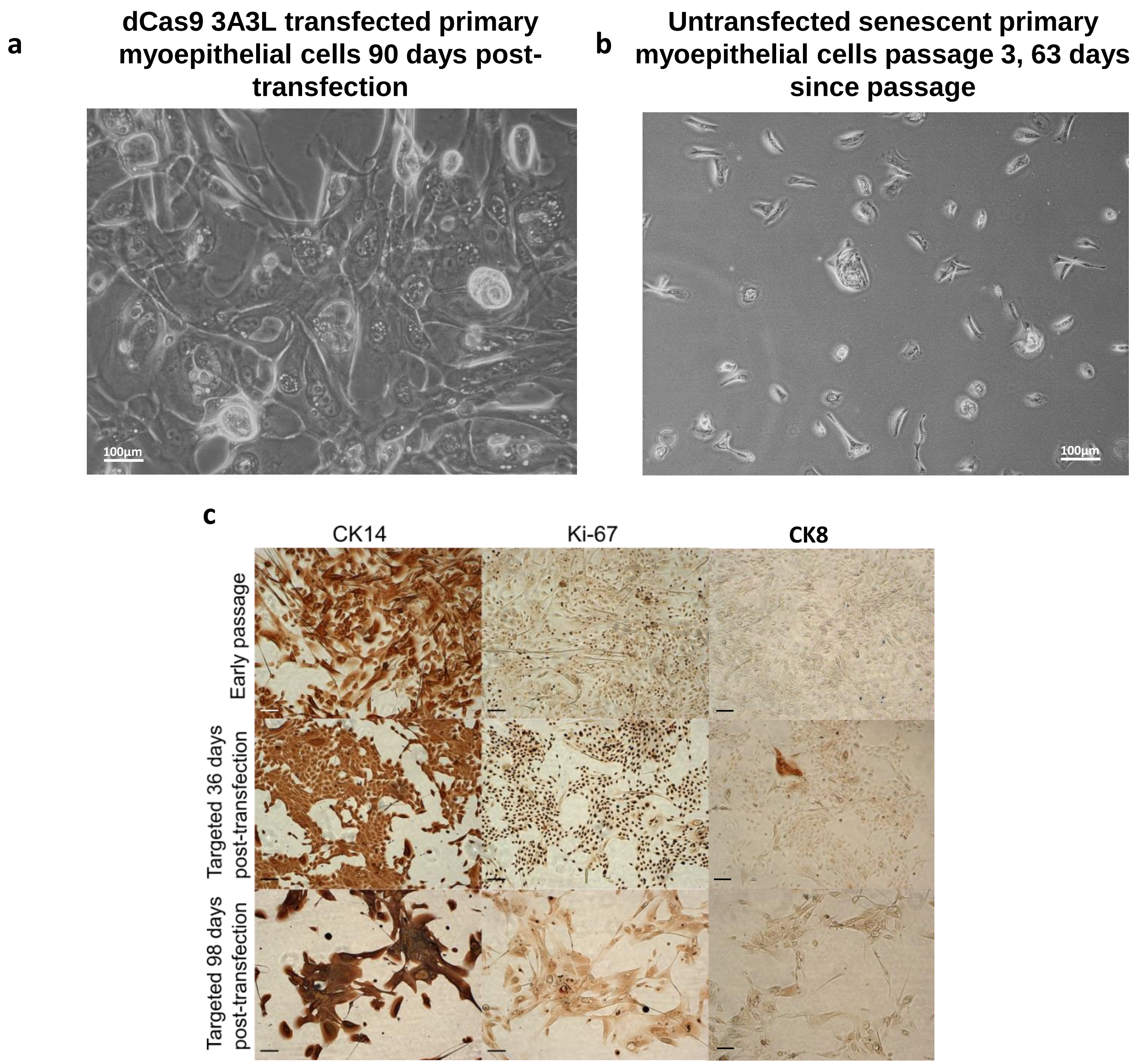

Supplementary Figure 8. Proliferating edited cells remain myoepithelial in lineage. (a) Primary myoepithelial cells from donor 1, 90 days post-transfection with 26 gRNAs (targeting *HIC1*, *RASSF1*, *PTEN* and *CDKN2A*), dCas9 3A3L and pMACS (cells were magnetically sorting at day 2). (b) Untransfected senescent primary myoepithelial cells from donor 1 after 65 days in culture. Cells were thawed at passage two and were passaged once before being maintained for a further 63 days. The media was replaced once a week. (c) Immunocytochemistry staining of donor 1 early passage untransfected primary myoepithelial cells (top row), dCas9 3A3L transfected cells (targeting *HIC1*, *RASSF1*, *PTEN* and *CDKN2A*), 36 days post-transfection (middle row) and dCas9 3A3L transfected cells (targeting *HIC1*, *RASSF1*, *PTEN* and *CDKN2A*) 98 days after transfection (bottom row). Cells were stained for CK14 (left column), Ki-67 (middle column) and CK8 (right column). Bars represent 100 µm.

a

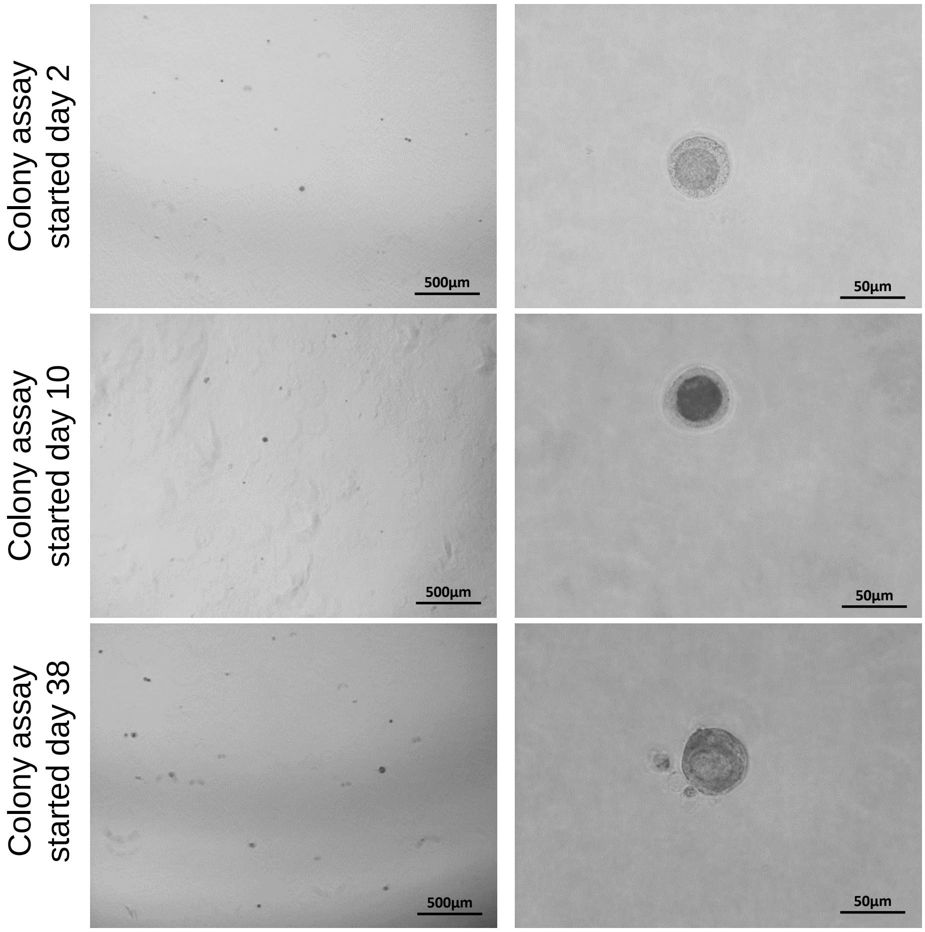

b

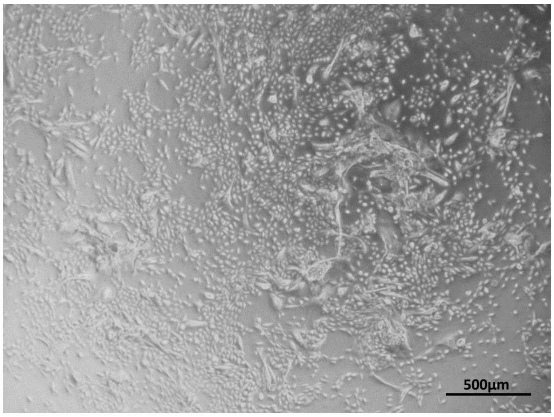

Supplementary Figure 9. Outgrowing myoepithelial cells are not forming 3D structures. (a) Light microscopy images taken 14 days after seeding primary myoepithelial cells from donor 1 into matrigel at various timepoints post-transfection with dCas9 3A3L (targeting *HIC1*, *RASSF1*, *PTEN* and *CDKN2A*) as specified to the left of the images. Bars represent 500 µm (left images) or 50 µm (right images). (b) Light microscopy image of cells rescued from 3D matrigel after 2 weeks and reseeded in a 2D culture plate (34 days post-transfection, 10 days after rescue from matrigel). Bar represents 500 µm.

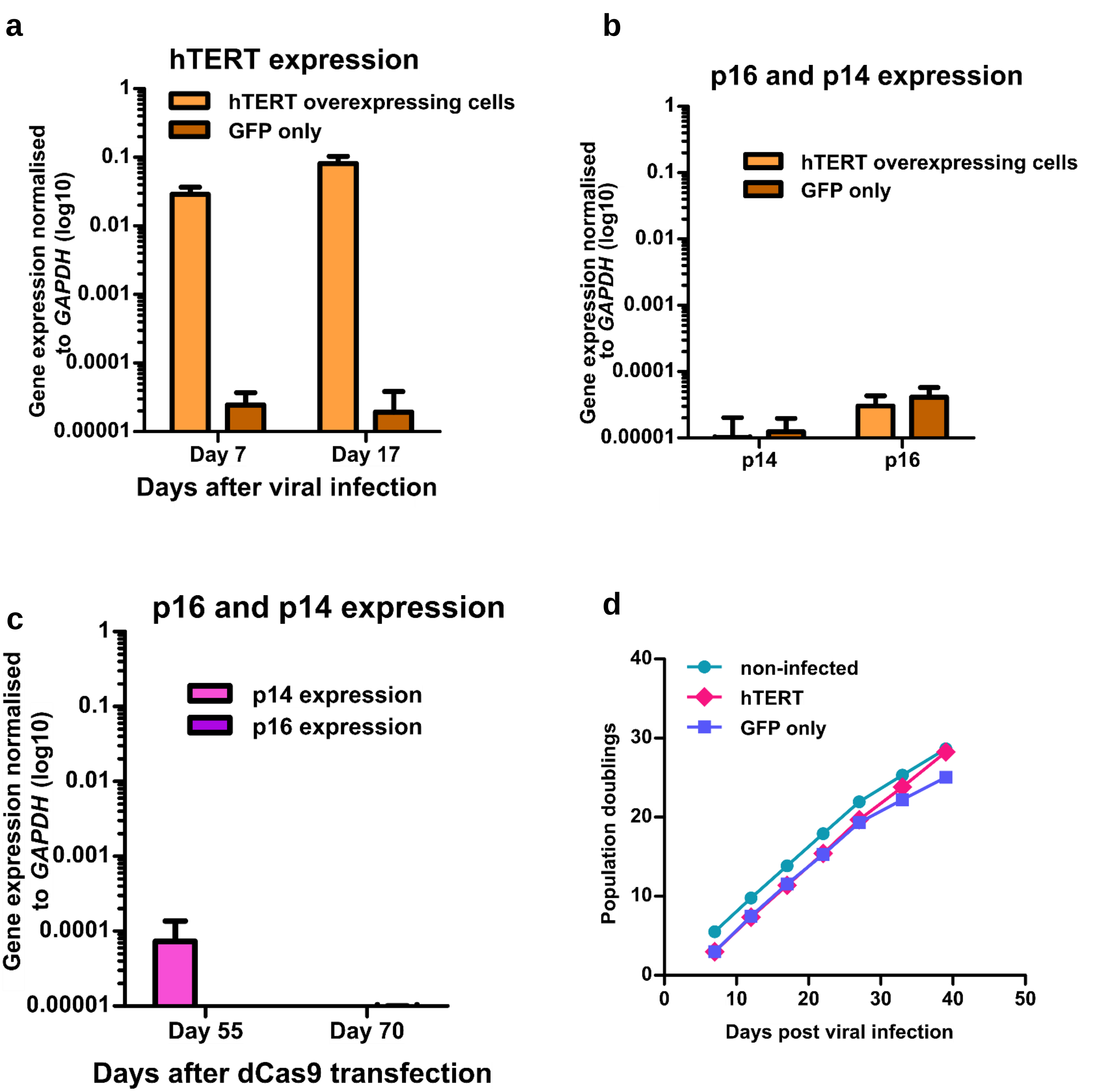

Supplementary Figure 10. hTERT overexpression and p14/p16 expression in outgrowing myoepithelial cells. (a) Gene expression measured by qPCR of hTERT, 7 and 17 days after infection in primary myoepithelial cells from donor 1. Primary cells were transiently transfected with dCas9 3A3L and gRNAs (targeting *HIC1*, *RASSF1*, *PTEN* and *CDKN2A*) and on day 38 post-transfection the cells were infected with a retrovirus containing a hTERT overexpressing vector (orange) or a GFP only expressing vector (brown). Gene expression is relative to *GAPDH* (mean ± SEM). (b) Gene expression measured by qPCR of p14 and p16 in hTERT overexpressing cells shown in (a) at 17 days post-infection. Gene expression is relative to *GAPDH* (mean ± SEM). (c) Gene expression measured by qPCR of p14 (pink) and p16 (purple) in primary myoepithelial cells from donor 1, 55 and 70 days after transfection with dCas9 3A3L and gRNAs (targeting *HIC1*, *RASSF1*, *PTEN* and *CDKN2A*). Gene expression is relative to *GAPDH* (mean ± SEM). (d) Cumulative population doublings over time from primary myoepithelial cells from donor 1 transiently transfected with dCas9 3A3L and 26 gRNAs (targeting *HIC1*, *RASSF1*, *PTEN* and *CDKN2A*) and then infected at day 38 with either no virus (green), hTERT overexpressing vector (pink) or a GFP only control (blue). Fifty thousand cells were seeded at the start of each passage and cells counted after 5 days (mean ± SEM, n = 3, where error bars are smaller than points plotted they are not shown).

PCA analysis of global DNA methylation from all samples measured from EPIC array

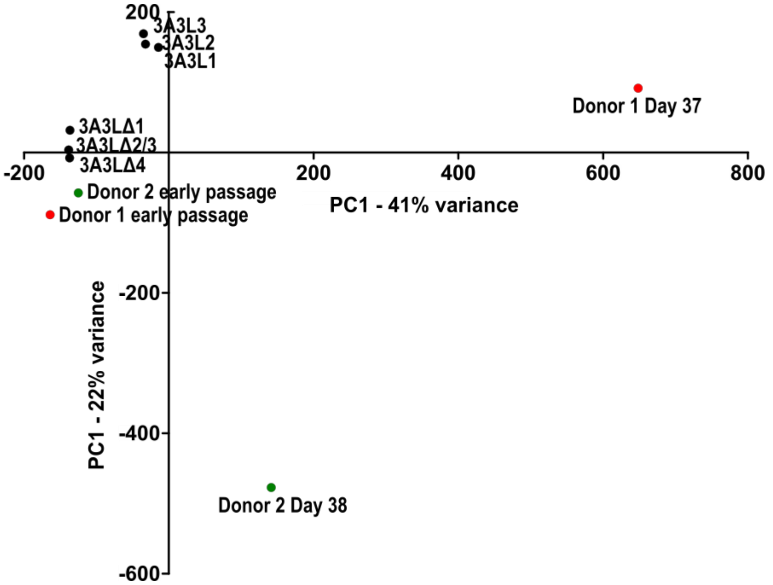

Supplementary Figure 11. PCA analysis of DNA methylation data from all probes in the EPIC array. Analysis was performed using data from: early passage primary myoepithelial cells (donor 1 and 2); donor 1 and 2 cells 37 and 38 days post-transfection respectively with dCas9 3A3L (targeting DNA methylation to *HIC1*, *RASSF1*, *PTEN* and *CDKN2A*); donor 1 primary myoepithelial cells 10 days post-transfection with dCas9 3A3L or 3A3LΔ (targeting DNA methylation to *HIC1*, *RASSF1*, *PTEN* and *CDKN2A*). All replicates are shown on the graph.

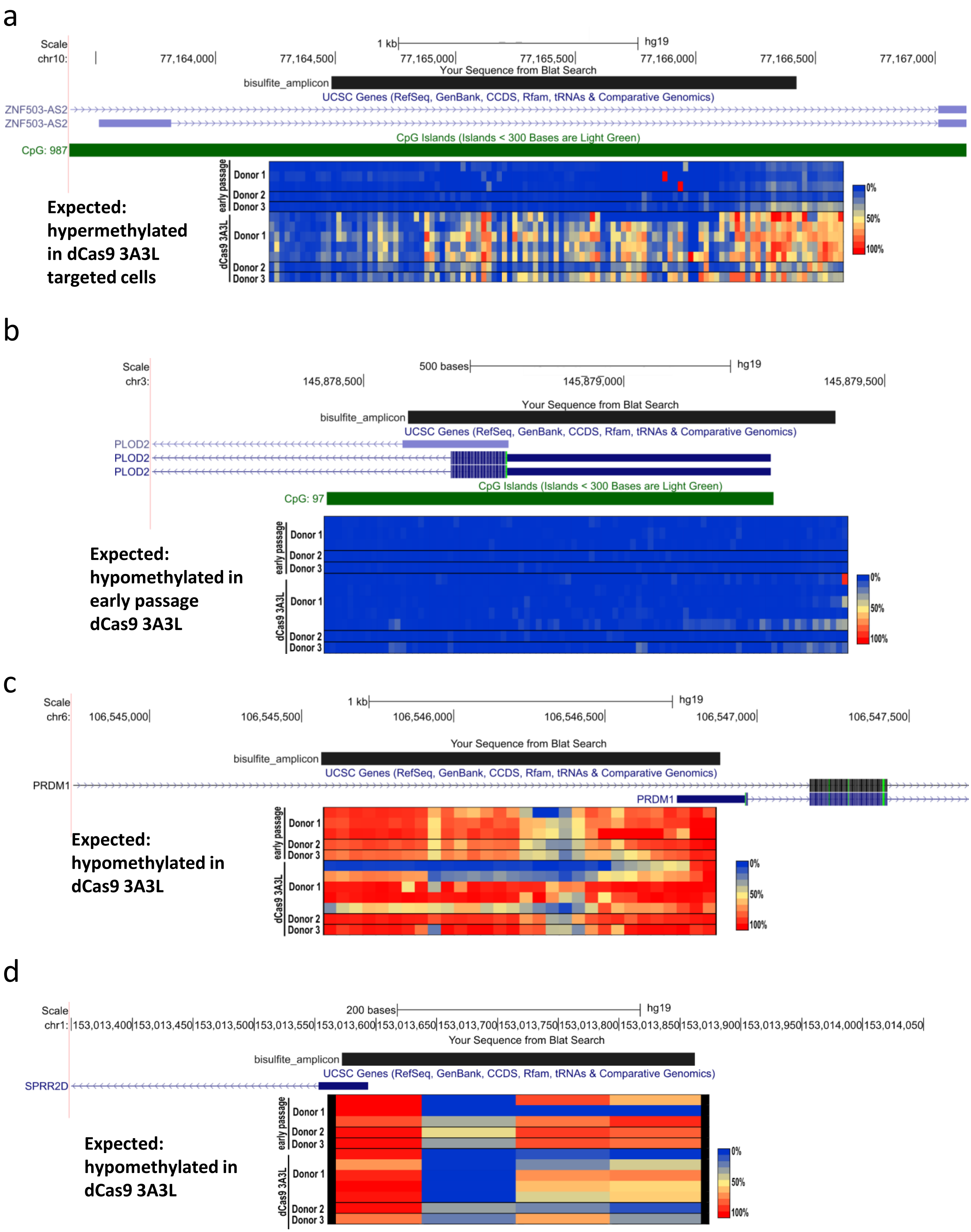
a
Scale
chr10:
1 kb
hg19
77,164,000
77,164,500
77,165,000
77,165,500
77,166,000
77,166,500
77,167,000
Your Sequence from Blat Search
bisulfite_amplicon
UCSC Genes (RefSeq, GenBank, CCDS, Rfam, tRNAs & Comparative Genomics)
ZNF503-AS2
ZNF503-AS2
CpG Islands (Islands < 300 Bases are Light Green)
CpG: 987
early passage
Donor 1
Donor 2
Donor 3
dCas9 3A3L
Donor 1
Donor 2
Donor 3
0%
50%
100%
Expected: hypermethylated in dCas9 3A3L targeted cells
b
Scale
chr3:
500 bases
hg19
145,878,500
145,879,000
145,879,500
Your Sequence from Blat Search
bisulfite_amplicon
UCSC Genes (RefSeq, GenBank, CCDS, Rfam, tRNAs & Comparative Genomics)
PLOD2
PLOD2
PLOD2
CpG Islands (Islands < 300 Bases are Light Green)
CpG: 97
early passage
Donor 1
Donor 2
Donor 3
dCas9 3A3L
Donor 1
Donor 2
Donor 3
0%
50%
100%
Expected: hypomethylated in early passage dCas9 3A3L
c
Scale
chr6:
1 kb
hg19
106,545,000
106,545,500
106,546,000
106,546,500
106,547,000
106,547,500
Your Sequence from Blat Search
bisulfite_amplicon
UCSC Genes (RefSeq, GenBank, CCDS, Rfam, tRNAs & Comparative Genomics)
PRDM1
PRDM1
early passage
Donor 1
Donor 2
Donor 3
dCas9 3A3L
Donor 1
Donor 2
Donor 3
0%
50%
100%
Expected: hypomethylated in dCas9 3A3L
d
Scale
chr1:
200 bases
hg19
153,013,400
153,013,450
153,013,500
153,013,550
153,013,600
153,013,650
153,013,700
153,013,750
153,013,800
153,013,850
153,013,900
153,013,950
153,014,000
153,014,050
Your Sequence from Blat Search
bisulfite_amplicon
UCSC Genes (RefSeq, GenBank, CCDS, Rfam, tRNAs & Comparative Genomics)
SPRR2D
early passage
Donor 1
Donor 2
Donor 3
dCas9 3A3L
Donor 1
Donor 2
Donor 3
0%
50%
100%
Expected: hypomethylated in dCas9 3A3L

Supplementary Figure 12. Further validation of EPIC data. Targeted bisulfite sequencing at (a) *C10orf41/ZNF503-AS2*, (b) *PLOD2*, (c) *PRDM1* and (d) *SPRR2D*. The localisation of the bisulfite amplicon (black bar) in relation to CGI (green bar) and TSS of genes is shown. Each rectangle represents the methylation % indicated by the colour key of a single CpG and each line is data from a single replicate. The top 5 lines show data from early passage (passage 2) primary myoepithelial cells from donors 1, 2 and 3, the bottom 7 lines show data from primary cells 37 – 41 days after transfection with dCas9 3A3L targeting *HIC1*, *RASSF1*, *PTEN* and *CDKN2A*.

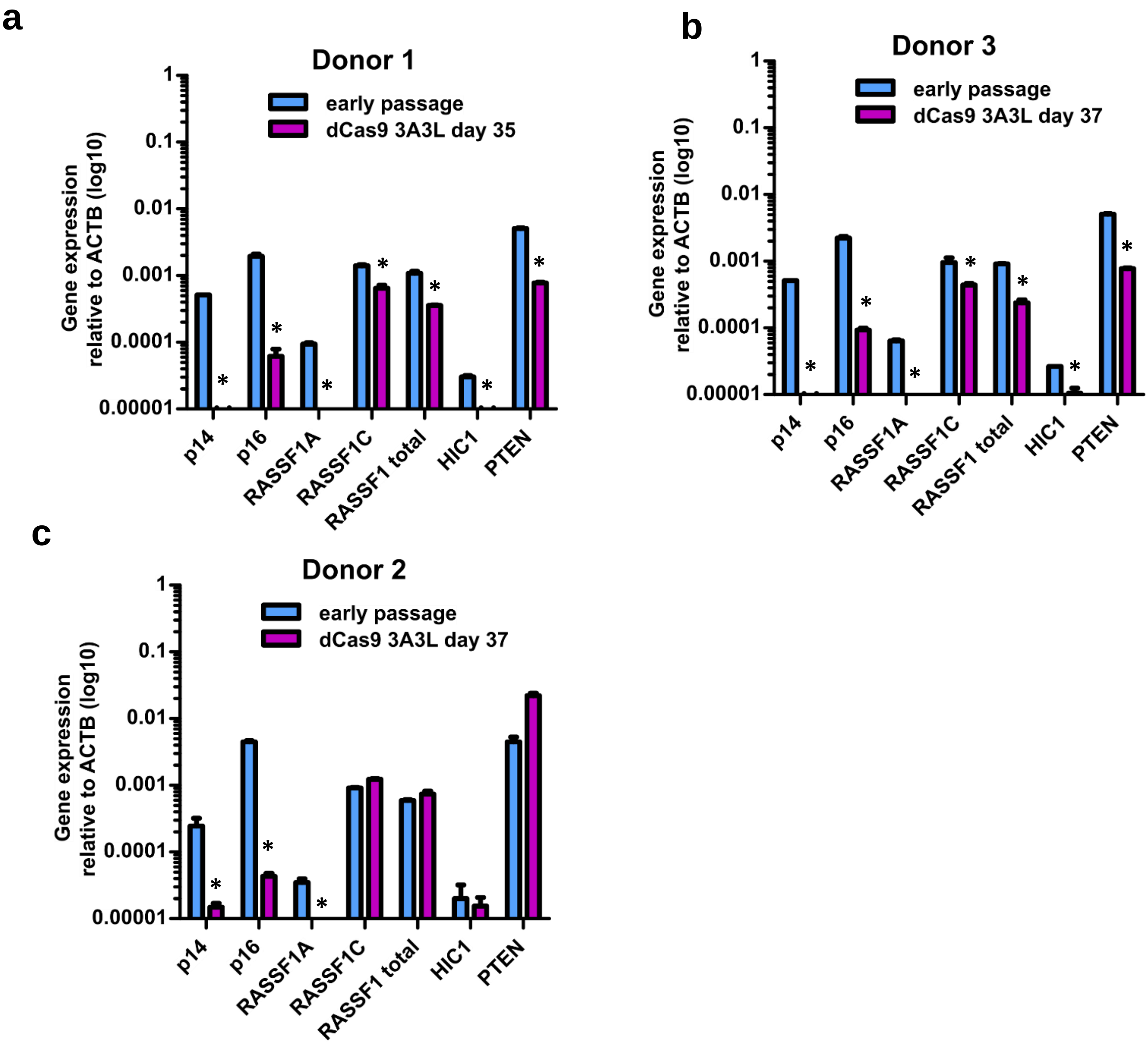

Supplementary Figure 13. Gene expression of target genes in early passage vs. dCas9 3A3L (35-37 days post-transfection) in three donors. (a) Gene expression of target genes in donor 1 early passage (passage 2) primary myoepithelial cells (blue) and dCas9 3A3L targeted cells 35 days post-transfection (pink). Gene expression is relative to *ACTB* (mean ± SEM). Statistical analysis: (two-way ANOVA): effect of gene, $F_{(6, 21)}$ = 942, p < 0.0001; effect of timepoint, $F_{(1,21)}$ = 2289, p < 0.0001; interaction gene x timepoint, $F_{(6,21)}$ = 528, p = 0.0001. Bonferroni post-hoc tests; *, p < 0.0001 compared to early passage cells. (b) Gene expression of target genes in donor 3 early passage (passage 2) primary myoepithelial cells (blue) and dCas9 3A3L targeted cells 35 days post-transfection (pink). Gene expression is relative to *ACTB* (mean ± SEM). Statistical analysis: (two-way ANOVA): effect of gene, $F_{(6, 21)}$ = 885, p < 0.0001; effect of timepoint, $F_{(1,21)}$ = 2055, p < 0.0001; interaction gene x timepoint, $F_{(6,21)}$ = 514, p = 0.0001. Bonferroni post-hoc tests; *, p < 0.0001 compared to early passage cells. (c) Donor 2 early passage (passage 2) primary myoepithelial cells (blue) and dCas9 3A3L targeted cells 35 days post-transfection (pink). Gene expression is relative to *ACTB* (mean ± SEM). Statistical analysis: (two-way ANOVA): effect of gene, $F_{(6, 21)}$ = 91, p < 0.0001; effect of timepoint, $F_{(1,21)}$ = 25, p < 0.0001; interaction gene x timepoint, $F_{(6,21)}$ = 49, p = 0.0001. Bonferroni post-hoc tests; *, p < 0.01 compared to early passage cells.

a

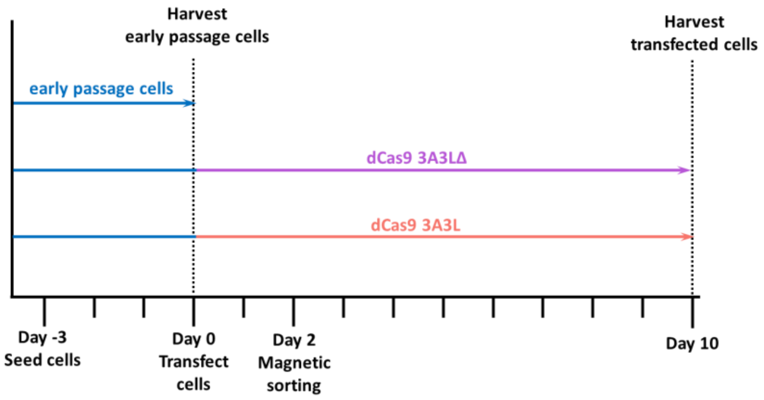

Supplementary Figure 14. Schematic showing the experimental protocol for the RNA-seq experiment. Early passage cells (blue) were harvested 3 days after seeding; while other cells were transfected with dCas9 3A3L (orange line) or 3A3LΔ (purple line), 26 gRNAs (targeting *HIC1*, *RASSF1*, *PTEN* and *CDKN2A*) and pMACS. Transfected cells were magnetically enriched at day 2 and harvested 10 days post-transfection.

a

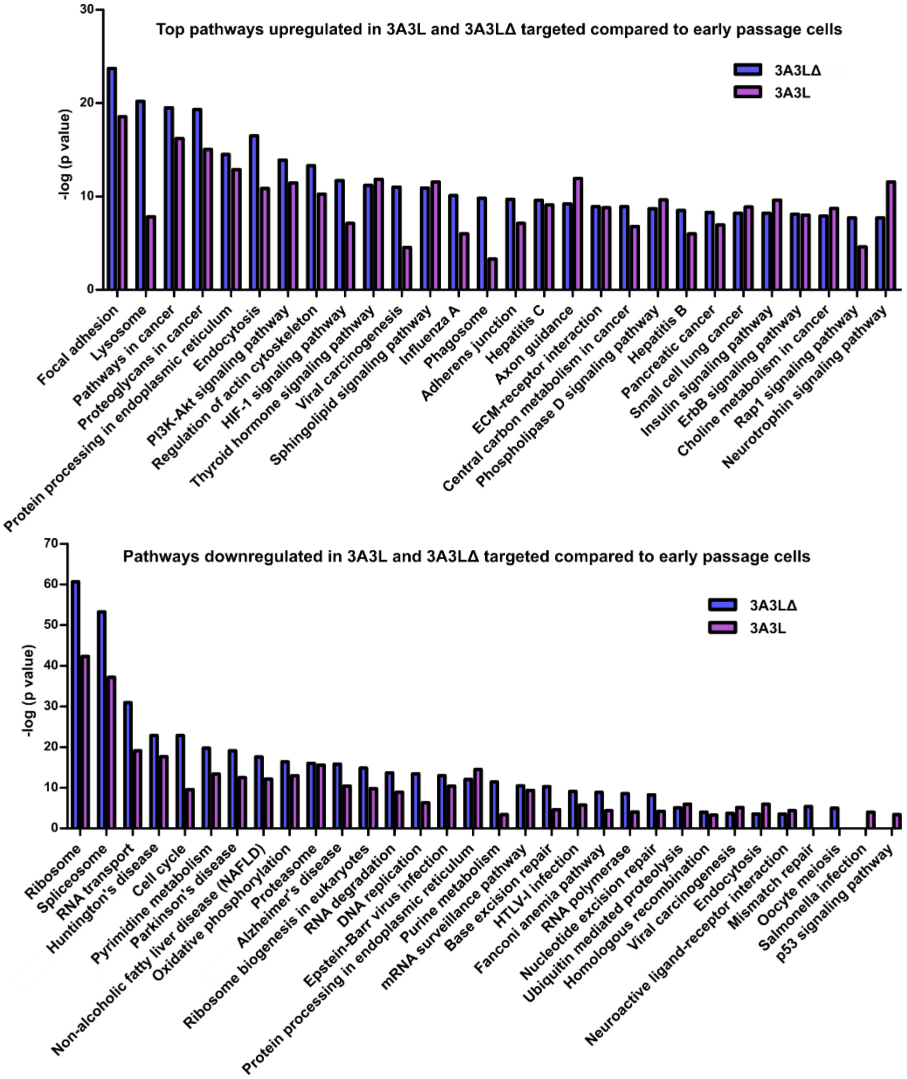
Top pathways upregulated in 3A3L and 3A3LΔ targeted compared to early passage cells
-log (p value)
0
10
20
30
3A3LΔ
3A3L
Focal adhesion
Lysosome
Pathways in cancer
Proteoglycans in cancer
Protein processing in endoplasmic reticulum
Endocytosis
PI3K-Akt signaling pathway
Regulation of actin cytoskeleton
HIF-1 signaling pathway
Thyroid hormone signaling pathway
Viral carcinogenesis
Sphingolipid signaling pathway
Influenza A
Phagosome
Adherens junction
Hepatitis C
Axon guidance
ECM-receptor interaction
Central carbon metabolism in cancer
Phospholipase D signaling pathway
Hepatitis B
Pancreatic cancer
Small cell lung cancer
Insulin signaling pathway
ErbB signaling pathway
Choline metabolism in cancer
Rap1 signaling pathway
Neurotrophin signaling pathway
Pathways downregulated in 3A3L and 3A3LΔ targeted compared to early passage cells
-log (p value)
0
10
20
30
40
50
60
70
3A3LΔ
3A3L
Ribosome
Spliceosome
RNA transport
Huntington's disease
Cell cycle
Pyrimidine metabolism
Parkinson's disease
Non-alcoholic fatty liver disease (NAFLD)
Oxidative phosphorylation
Proteasome
Alzheimer's disease
Ribosome biogenesis in eukaryotes
RNA degradation
DNA replication
Epstein-Barr virus infection
Protein processing in endoplasmic reticulum
Purine metabolism
mRNA surveillance pathway
Base excision repair
HTLV-I infection
Fanconi anemia pathway
RNA polymerase
Nucleotide excision repair
Ubiquitin mediated proteolysis
Homologous recombination
Viral carcinogenesis
Endocytosis
Neuroactive ligand-receptor interaction
Mismatch repair
Oocyte meiosis
Salmonella infection
p53 signaling pathway

b

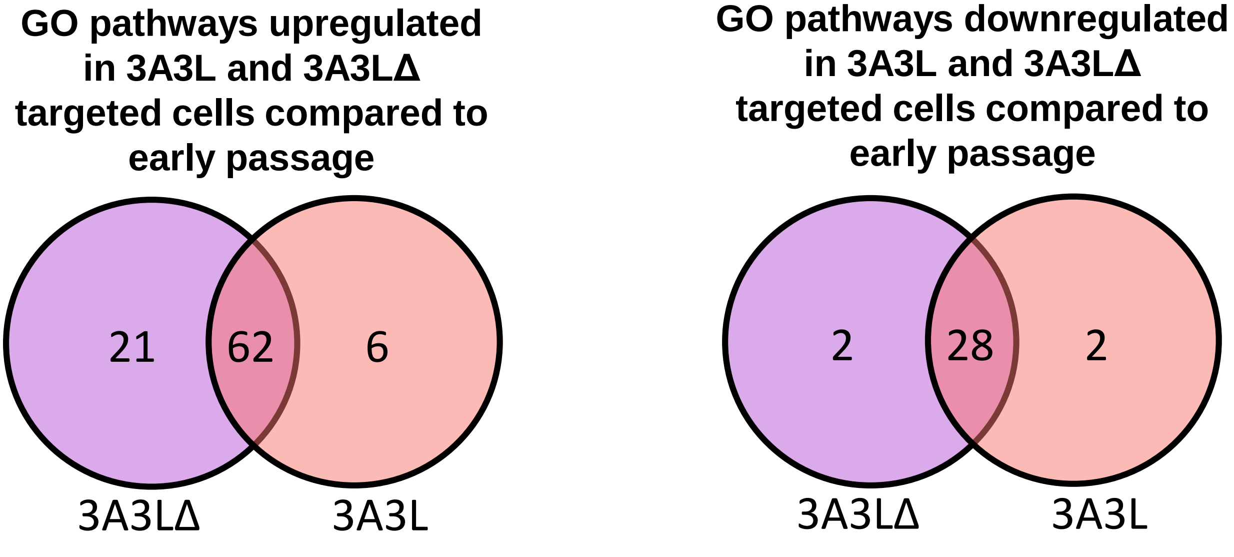
GO pathways upregulated in 3A3L and 3A3LΔ targeted cells compared to early passage
21
62
6
3A3LΔ
3A3L
GO pathways downregulated in 3A3L and 3A3LΔ targeted cells compared to early passage
2
28
2
3A3LΔ
3A3L

Supplementary Figure 15. Pathway analysis of the RNA-seq data. (a; top panel) Graph using RNA-seq data and GO analysis to show the top pathways upregulated in dCas9 3A3LΔ (blue) and dCas9 3A3L (purple) targeted cells 10 days post-transfection compared to the early passage cells. Data displayed as –log(p value), cells were transfected with dCas9 3A3L or 3A3LΔ targeting *HIC1*, *RASSF1*, *PTEN* and *CDKN2A* and pMACS. (a; bottom panel) Graph using RNA-seq data and GO analysis to show the pathways downregulated in dCas9 3A3LΔ (blue) and dCas9 3A3L (purple) targeted cells 10 days post-transfection compared to the early passage cells. Data displayed as –log(p value), cells were transfected with dCas9 3A3L or 3A3LΔ targeting *HIC1*, *RASSF1*, *PTEN* and *CDKN2A* and pMACS. (b; left panel) Overlap between GO pathways upregulated in dCas9 3A3LΔ and dCas9 3A3L targeted cells compared to early passage cells. (b; right panel) Overlap between GO pathways downregulated in dCas9 3A3LΔ and dCas9 3A3L targeted cells compared to early passage cells.

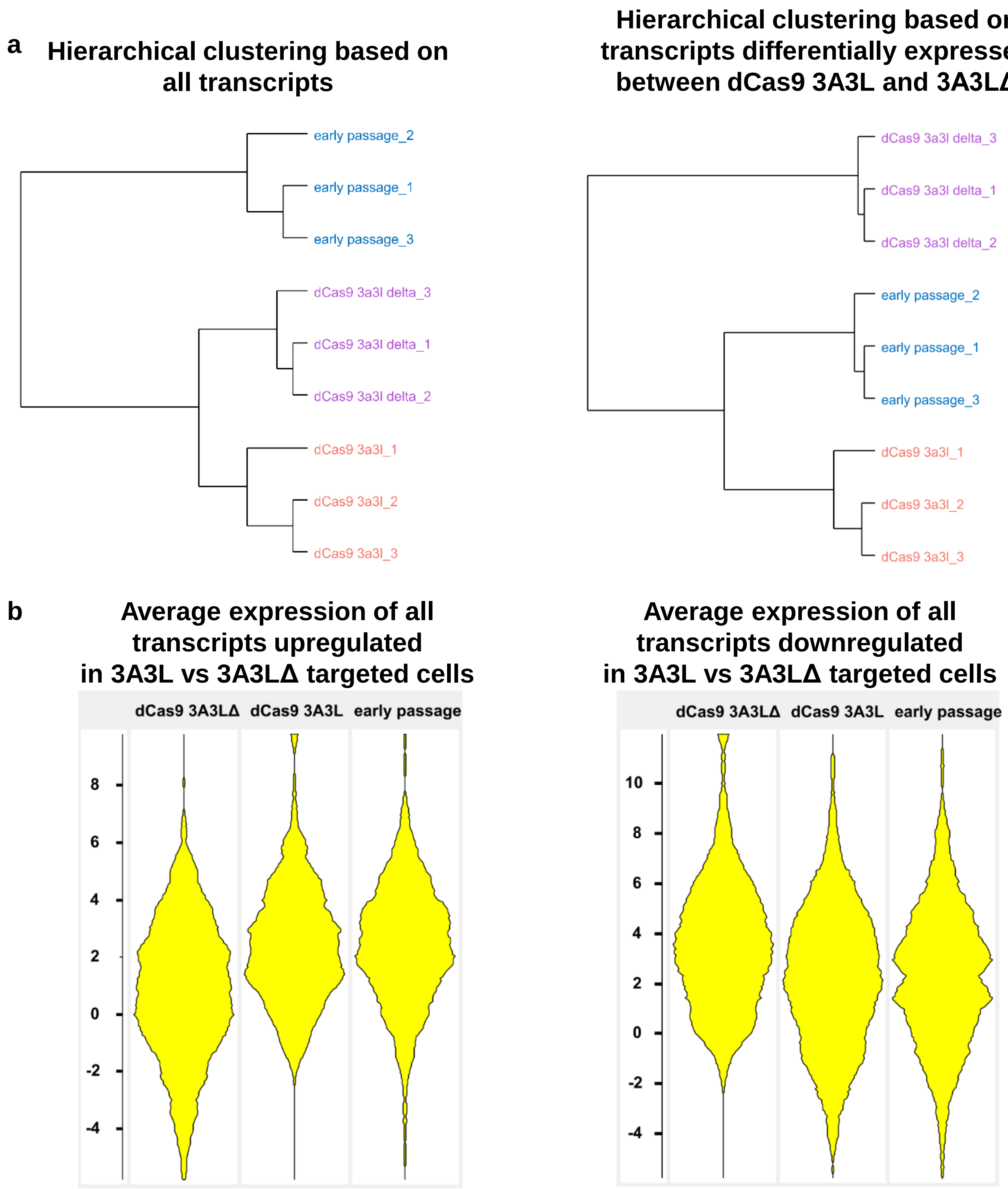

Supplementary Figure 16. Hierarchical clustering of RNA-seq data. (a; left panel) hierarchical clustering using total RNA-seq transcripts from early passage (blue), 3A3LΔ (purple) and 3A3L (orange) targeted cells 10 days-post transfection (targeted to *HIC1*, *RASSF1*, *PTEN* and *CDKN2A*). (a: right panel) hierarchical clustering using subset of transcripts that are differentially expressed between 3A3LΔ (purple) and 3A3L (orange) targeted cells 10 days post-transfection and the corresponding level of expression of these transcripts in early passage cells (blue). (b; left panel) violin plot showing average expression of all transcripts that are upregulated in 3A3L compared to 3A3LΔ targeted cells 10 days post-transfection, and the expression of those genes in early passage cells. (b; right panel) violin plot showing average expression of all transcripts that are downregulated in 3A3L compared to 3A3LΔ targeted cells 10 days post-transfection, and the expression of those genes in early passage cells. Data was analysed and graphs generated using SeqMonk.

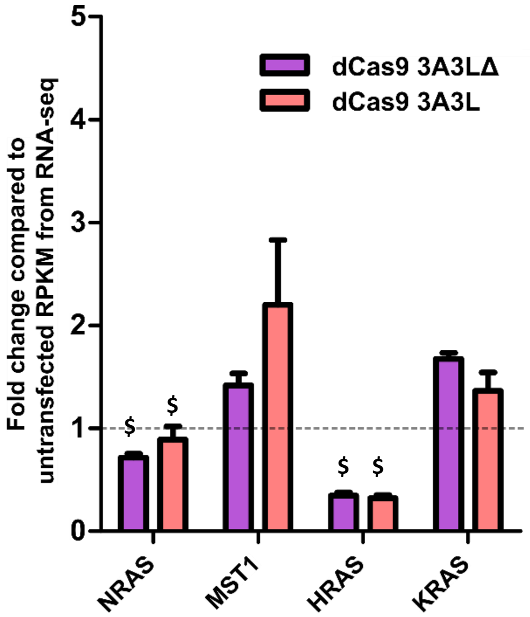

Supplementary Figure 17. RASSF1 downstream gene expression. Graph plotting the RPKM values from the RNA-seq data of transcripts associated with RASSF1 signalling 10 days after transfection with dCas9 3A3L or 3A3LΔ, 26 gRNAs (targeting *HIC1*, *RASSF1*, *PTEN* and *CDKN2A*) and pMACS. Data is shown as fold change of 3A3LΔ (purple) and 3A3L (orange) RPKM data compared to early passage cells (dotted line; mean ± SEM, n = 3). Significance is from *DESeq2* analysis with Benjamini-Hochberg correction: $, $p < 0.001$, 3A3L or 3A3LΔ compared to early passage cells.

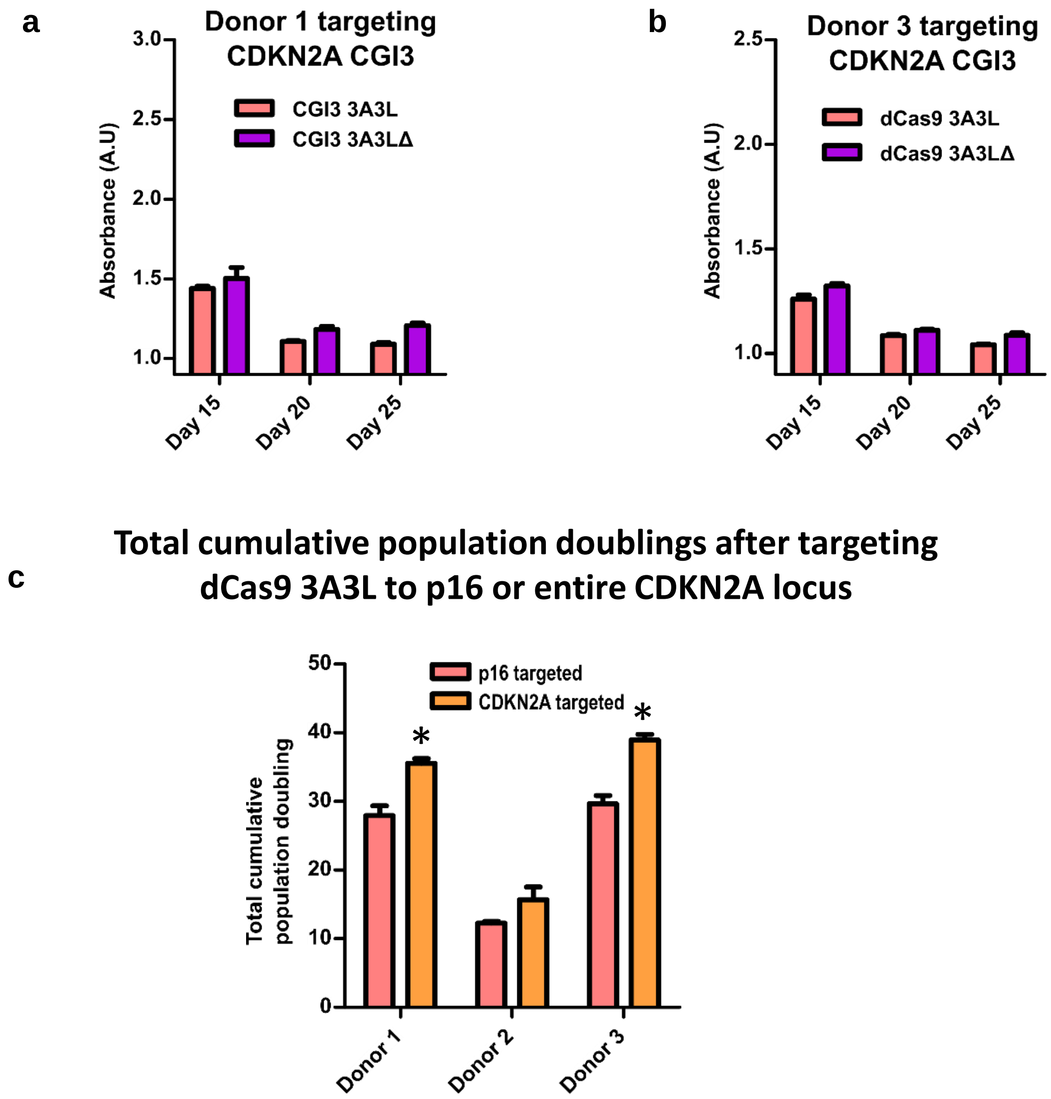

Supplementary Figure 18. Targeting the *CDKN2A* locus. (a – b) Proliferation was assessed using a colorimetric assay 15, 20 and 25 days after targeting *CDKN2A* CGI3 with dCas9 3A3L (orange) or dCas9 3A3LΔ (purple) without magnetic sorting using (a) donor 1 and (b) donor 2 primary myoepithelial cells. The data is shown as fold change compared to the average absorbance from 3 wells without cells (mean ± SEM, n = 3). (c) Total cumulative population doublings from myoepithelial cells from donors 1, 2 and 3 after targeting dCas9 3A3L to *HIC1*, *RASSF1*, *PTEN* and *CDKN2A* (mean ± SEM, n = 3). Statistical analysis: (two-way ANOVA): effect of gene targeting, $F_{(1, 12)} = 62$, $p < 0.0001$; effect of donor, $F_{(2,12)} = 353.8$, $p < 0.0001$; interaction gene targeted x donor, $F_{(2,12)} = 4.579$, $p = 0.03$. Bonferroni post-hoc tests; *, $p < 0.001$ compared to p16 targeted cells.

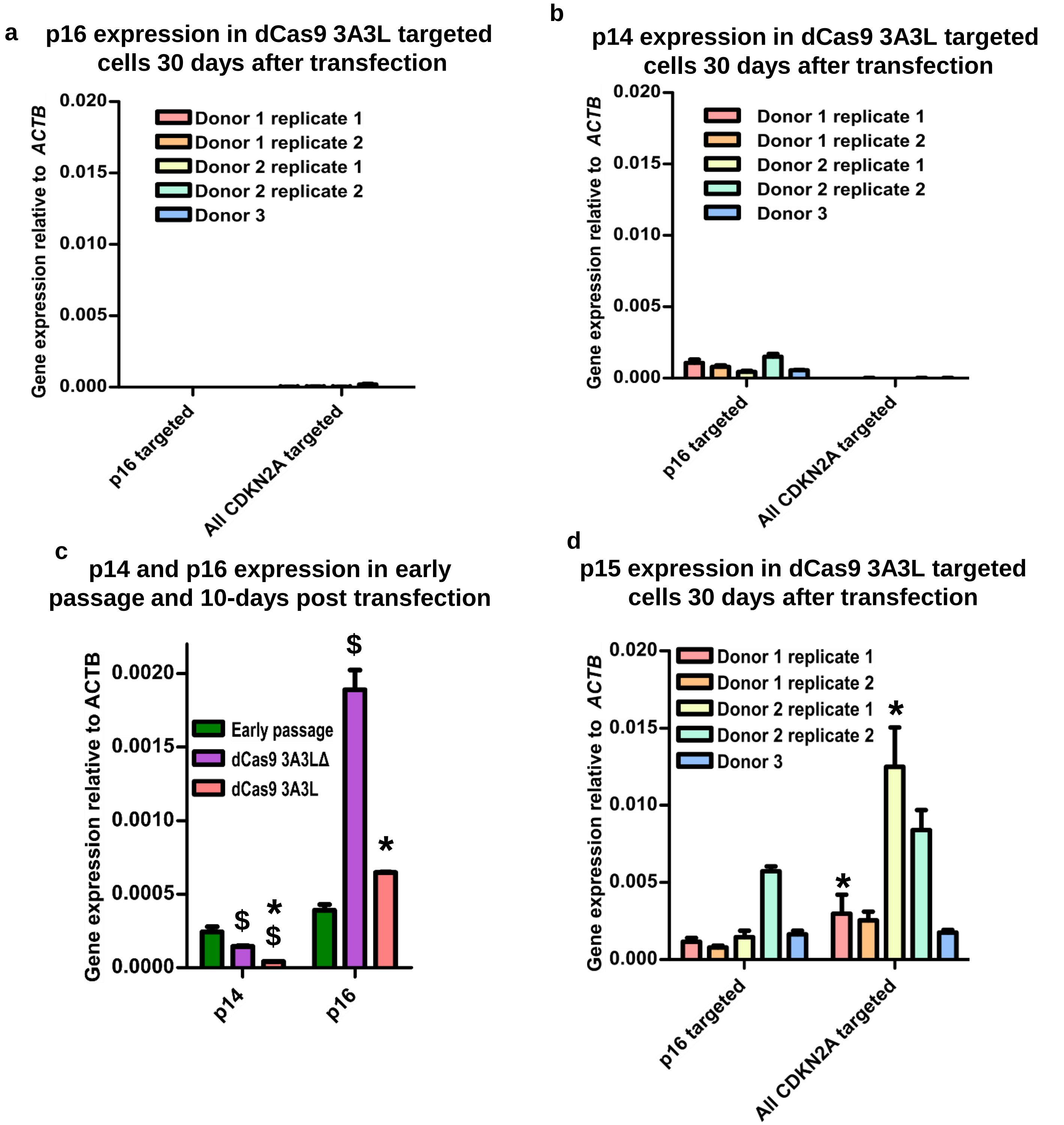

Supplementary Figure 19. p14, p15 and p16 expression in proliferating cells. Gene expression of (a) p16 and (b) p14 measured by qPCR in donor 1 (red and orange), donor 2 (yellow and green) and donor 3 (blue) 30 days post-transfection with dCas9 3A3L and gRNAs to either p16 (3 gRNAs) or CDKN2A (8 gRNAs). Expression is relative to *ACTB* (mean ± SEM) (c) Gene expression of p14 and p16 from early passage (green), dCas9 3A3LΔ (purple) and dCas9 3A3L (orange) transfected cells 10 days post-transfection targeting to *HIC1*, *RASSF1*, *PTEN* and *CDKN2A*. Expression is relative to *ACTB* (mean ± SEM; statistical analysis: two-tailed T-tests. *, p < 0.01 dCas9 3A3L compared to 3A3LΔ; $, p < 0.01 dCas9 3A3L or 3A3LΔ compared to early passage cells). (d) Gene expression of p15 measured by qPCR in donor 1 (red and orange), donor 2 (yellow and green) and donor 3 (blue) 30 days post-transfection with dCas9 3A3L and gRNAs to either p16 (3 gRNAs) or CDKN2A (8 gRNAs). Expression is relative to *ACTB* (mean ± SEM; statistical analysis: two-tailed T-tests; * p < 0.05 compared to p16 targeted cells).

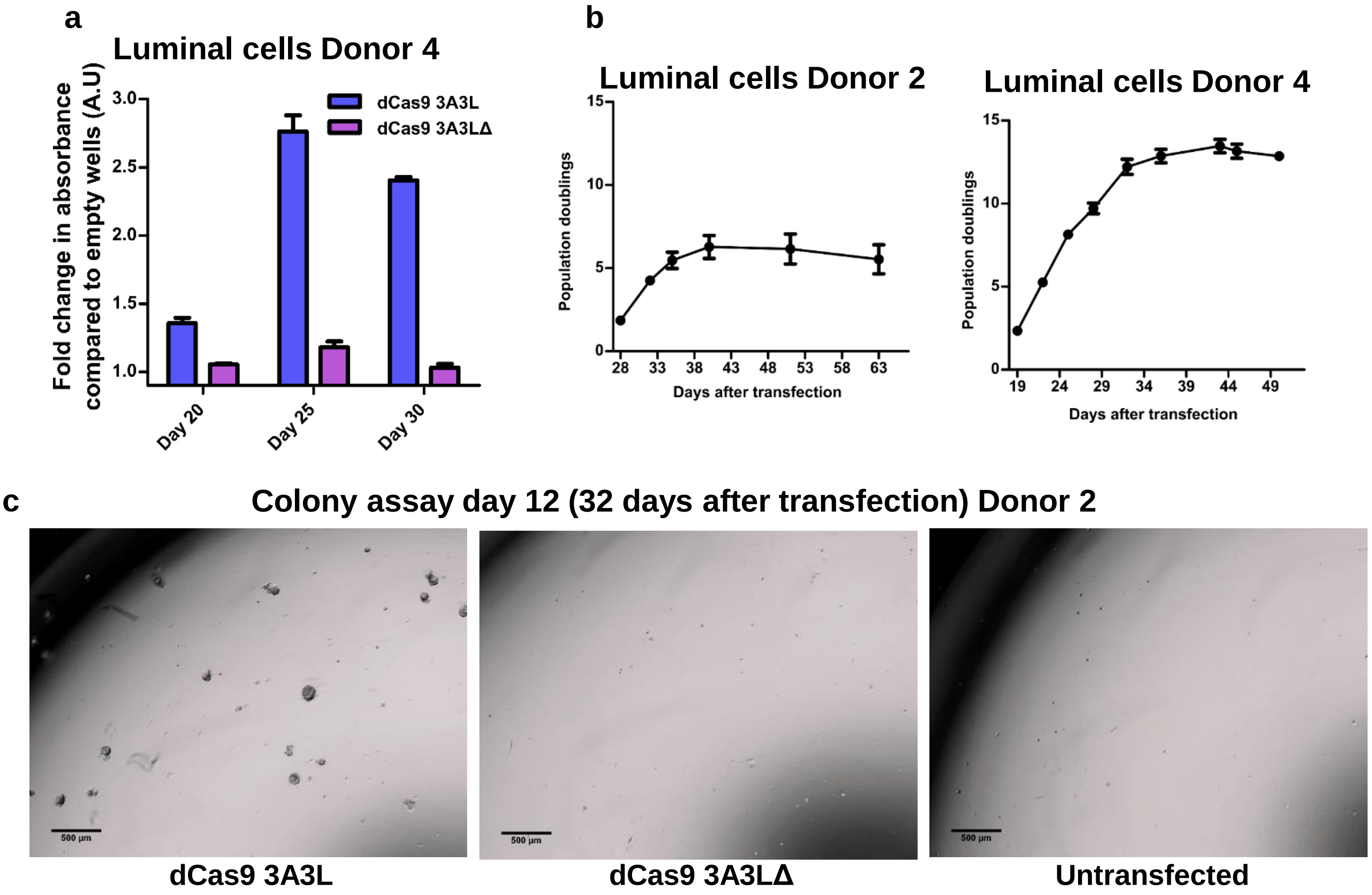

Supplementary Figure 20. Prevention of senescence entry and anchorage independent growth in luminal cells. (a) Proliferation was assessed using a colorimetric assay 20, 25 and 30 days after targeting *CDKN2A* with dCas9 3A3L (blue) or dCas9 3A3LΔ (purple) without magnetic sorting using donor 4 primary luminal cells. The data is shown as fold change compared to the average absorbance from 3 wells without cells (mean ± SEM, n = 3). (b) Cumulative population doublings over time from primary luminal cells from donor 2 (left graph) and donor 4 (right graph) transfected with dCas9 3A3L and 8 gRNAs (targeting *CDKN2A*). Fifty thousand cells were seeded at the start of each passage and cells counted after 2 – 3 days. Decreasing population doublings depict when fewer cells were counted at the end of passage compared to the start (mean ± SEM, n = 3, where error bars are smaller than points plotted they are not shown). (c) Light microscopy images taken 12 days after seeding primary luminal cells from donor 2 into matrigel and 32 days post-transfection with 8 gRNAs (targeting *CDKN2A*) and dCas9 3A3L (left panel), dCas9 3A3LΔ (middle panel) or untransfected cells (right panel). Bars represent 500 µm. Bar represents 500 µm.

Comparing the top 300 hypermethylated probes in dCas9 3A3L replicates from EPIC array (Average probe methylation in dCas9 3A3LΔ cells < 5%)

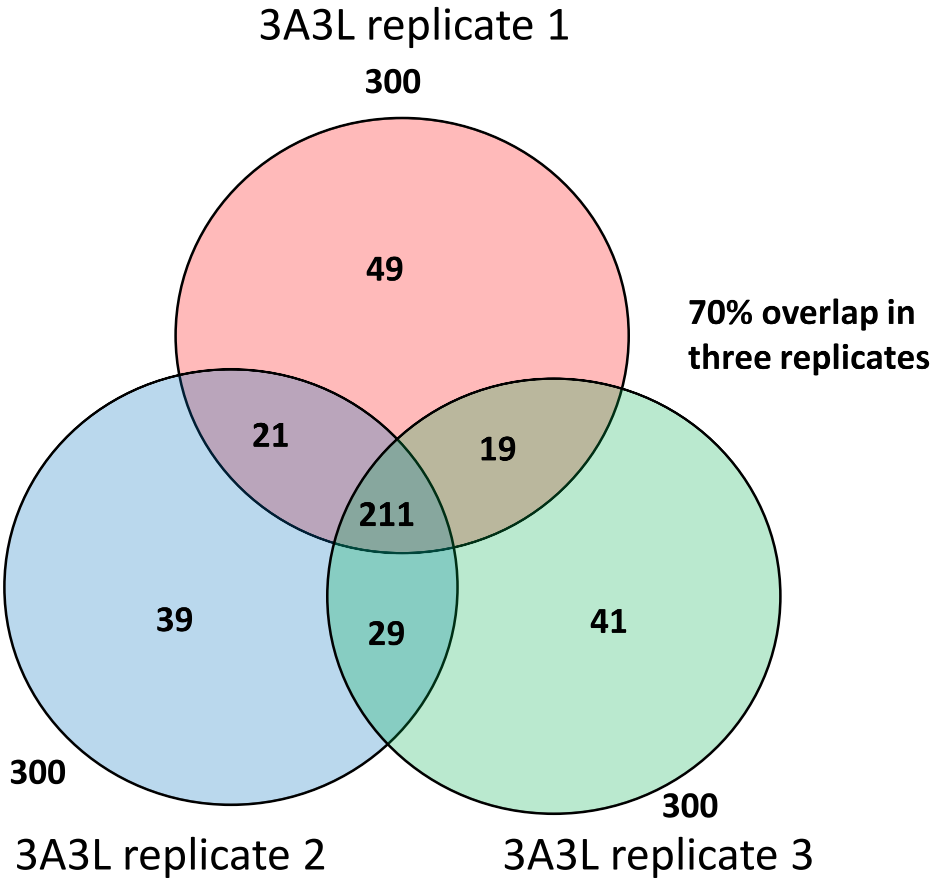

Supplementary Figure 21. Off-target methylation overlap between replicates. Venn diagram showing overlap between 300 probes with the greatest methylation increase in 3 independent replicates of dCas9 3A3L targeted cells, 10 days post-transfection from the EPIC array data (only probes 0 – 5% methylated in 3A3LΔ and > 20% in 3A3L targeted cells were selected). Primary myoepithelial cells from donor 1 were transfected with dCas9 3A3L or 3A3LΔ targeted *HIC1*, *RASSF1*, *PTEN* and *CDKN2A*. 10 days post-transfection (including magnetic sorting), cells were harvested and global DNA methylation analysed. Only the probes with 0 – 5% methylation on average in the 3A3LΔ samples were selected. Each 3A3L targeted replicate was assessed separately and the 300 probes with the greatest methylation increase were selected and compared.

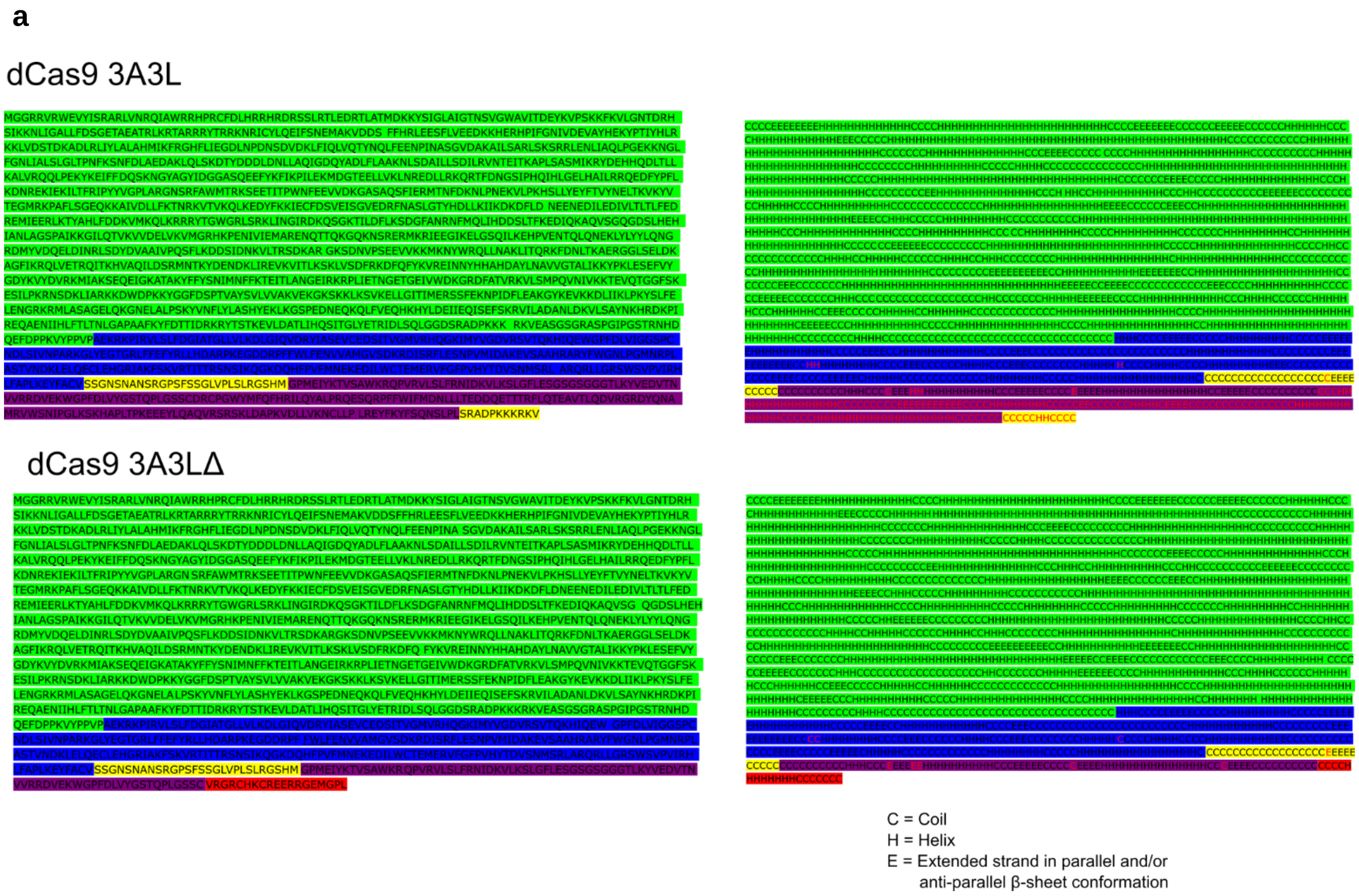

Supplementary Figure 22. dCas9 effector fusion sequences. (a) dCas9 3A3LΔ has disrupted folding of the 3A3L section of the protein, inhibiting methyltransferase activity, without affecting dCas9 folding.

Supplementary Table 1. gRNA sequences

All sequences 5’ – 3’ orientation, protospacer adjacent motif (PAM) sequence underlined

Target	gRNA number	Sequence (5' - 3')
HIC1	1	GACTCGTCGTGGGTTCCTTTAGG
	2	CGAATCCATGCGCCTGAGTGAGG
	3	TCCCCGTAGACATTACTGTACGG
	4	GTGCCTAGAAATACGTACATGGG
	5	ATAGCCGCAGCAGCGCCCGCCGG
RASSF1 CGI 1	1	TTGCTTCAGCAAACCGGACCAGG
	2	GCACCCAGGTTTCCATTGCGCGG
	3	GCTTTGGGCGCTAGCAAGCGCGG
	4	TGGCACCCGCTGGGCGCGCTGGG
	5	TGCAGTGCGCGCGTGAGTAGTGG
RASSF1 CGI 2	1	ATTAGAACGCTCCTTGCGCGCGG
	2	TTGTGCGCTTGCCCGGACGCTGG
	3	CGTACACGCACGCACGCGACCGG
	4	GACGCGGCAACGGACCGGGGAGG
PTEN	1	CGGGCGCCTCGGAAGACCGAGGG
	2	GCTGCGGCAGGATACGCGCTCGG
	3	AGCGCCTGTGAGCAGCCGCGGGG
	4	TTAAAACCGGCCCGGGTCCCTGG
CDKN2A CGI 1 (p14)	1	GCGGGAAAGTGGCGGTAGGCGGG
	2	TCAGAGCCGTTCCGAGATCTTGG
	3	CTGCCCCCTTAACTGCAGACTGG
	4	GCACGCGCGCCGAATCCGGAGGG
CDKN2A CGI 1 (p16)	1	CGCCAGAGCCAGCGTTGGCAAGG
	2	AGGGACCGCGGTATCTTTCCAGG
	3	GCATGGAGCCTTCGGCTGACTGG
CDKN2A CGI 3	1	TCCCGGGCAGCGTCGTGCACGGG

Supplementary Table 2. bisulfite cloning and targeted bisulfite sequencing primers

Target	Name	Sequence (5' - 3')	Target	Name	Sequence (5' - 3')
HIC1	bis_cloning_1_F	GAATTGGGGTTGTGTGTTTAG	p14 (CDKN2A)	bis_seq_1_F	ATTTTTGAGGTGGGTTTAGAAGTTT
	bis_cloning_1_R	ATGTTTTTTAGGTGGGTTTT		bis_seq_1_R	CTAAAAAACCAAATAAAAAAAAACCCT
HIC1	bis_seq_1_F	GGGTTTTATGGTTATTTTTTTGGAT		bis_seq_2_F	GAGGGTTTTTTTTATTTGGTTTTTT
	bis_seq_1_R	ACATAAACTAATTTATCCCCAACCC		bis_seq_2_R	TCCCAATCTACAATTAAAAAAACAAA
	bis_seq_2_F	GGGTTGGGGATAAATTAGTTTATGT		bis_seq_3_F	TTTGTTTTTTTAATTGTAGATTGGGA
	bis_seq_2_R	ATCCCAAAAACCCTAAAACTTTAAA		bis_seq_3_R	CTAAAAATAAAAAAATAATAATAAAAAT
	bis_seq_3_F	TTAAAGTTTTAGGGTTTTTGGGATT		bis_seq_4_F	ATTTTTATTATTATTTTTTTATTTTTAG
	bis_seq_3_R	AAAACCCACCTAAAAAACATATC		bis_seq_4_R	ACTAACATTCAACCTCCTAATTAAC
RASSF1A	bis_cloning_1_F	GTTTTATTTAGTGGGTAGGTTAAGTGTG	p16 (CDKN2A)	bis_seq_1_F	TTGATTTTAATTTTTTTGTAAATTT
	bis_cloning_1_R	CAACTCAATAAACTCAAACTCCCC		bis_seq_1_R	AAAAAAAACTAACTAATCACCAAAA
	bis_seq_1_F	TTTTTTATTTTAAAGGTTTTGGAGG		bis_seq_2_F	TTTTGGTGATTAGTTAGTTTTTTTT
	bis_seq_1_R	AAAAAAACAACCAAAAAACAAC		bis_seq_2_R	AAACCCAATCCTCCTTCCTTAC
	bis_seq_2_F	GTTGTTTTTTGGTTGTTTTTTT		bis_seq_3_F	GGTTTTTTTTTATTTGTTTTTTATAT
	bis_seq_2_R	AAAAAACCTAAACTCATTAAACTAC		bis_seq_3_R	AAAAAAATAATTTTACTTTTTCTTATAATT
	bis_seq_3_F	GTAGTTTAATGAGTTTAGGTTTTTT	C10orf41/ ZNF503-AS2	bis_seq_1_F	GGTAGTTTTAGTATTTGAAGTTTGGG
	bis_seq_3_R	AAAACCCTCTTCCTCTAACACAATAA		bis_seq_1_R	CAAAATAATTAATAACCCCCACTC
	bis_seq_4_F	TTATTGTGTTAGAGGAAGAGGGTTTT		bis_seq_2_F	GAGTGGGGGTTATTAATTATTTTG
	bis_seq_4_R	AACCCAAAATAACAAAACCAAATAA		bis_seq_2_R	TACTCTAAACCAAACTTTCCAACTC
RASSF1B and C	bis_seq_1_F	GGTTTTAGTTATAGTTGGATAATGTT		bis_seq_3_F	GAGTTGGAAAGTTTGGTTTAGAGTA
	bis_seq_1_R	TCCTACCCCAATAAAACAAAAACTA		bis_seq_3_R	AATCTAAACCTTACTCCCAATCAAA
	bis_seq_2_F	GGTATTTTTTGGTTTTTATTTGGTT		bis_seq_4_F	TTTGATTGGGAGTAAGGTTTAGATT
	bis_seq_2_R	TCCTACCCCAATAAAACAAAAACTA		bis_seq_4_R	AAAACTACACACTCCTAAAACAACC
	bis_seq_3_F	TAGTTTTTGTTTTATTGGGGTAGGA		bis_seq_5_F	GGTTTTTTGTTTTGGGATGTTT
	bis_seq_3_R	AACAAACCACAATACAAACATTCTC		bis_seq_5_R	AAACCCCATACACCATACAAATTAA
	bis_seq_4_F	GTTTGTATTGTGGTTTGTTTATTTT	PLOD2	bis_seq_1_F	TTTTTATTATTTATTTGGTTAGTTT
	bis_seq_4_R	CAAACCTCTCTACCTATAACCTTCC		bis_seq_1_R	TAAAACCTCAACTACTACTCCTAAC
	bis_seq_5_F	TGTTATTTAGGTTGGAGTGTAATGG		bis_seq_2_F	GTTAGGAGTAGTAGTTGAGGTTTTA
	bis_seq_5_R	AACAAATAACAACCACAAAACAAAC		bis_seq_2_R	CACTCCCAAAACTAAATACAAACTT
PTEN	bis_seq_1_F	TTAGATAGGTGTTTTTTGGGTTTTT		bis_seq_3_F	AAGTTTGTATTTAGTTTTGGGAGTG
	bis_seq_1_R	CCCCCAAATCTATATCCTCATAATAT		bis_seq_3_R	AAATCCCAATCAACCAAAATTAC
	bis_seq_2_F	ATATTATGAGGATATAGATTTGGGGG	PRDM1	bis_seq_1_F	TTTTGTGTGAGTGATTTTATGGTAT
	bis_seq_2_R	CAAACTCTACTAAACATACCCAATATA		bis_seq_1_R	AAAACTCTATCCCTTTAACACAAAC
	bis_seq_3_F	TATATTGGGTATGTTTAGTAGAGTTTG		bis_seq_2_F	TTTTATAAGTTGTTTTGATGAAGTTATATG
	bis_seq_3_R	AACTTCCATCATAACTACAACTTCC		bis_seq_2_R	CTCTTCCCCTCTTTTTAAAAAATAC
	bis_seq_4_F	GGAAGTTGTAGTTATGATGGAAGTT		bis_seq_3_F	ATTTTTTAAAAAGAGGGGAAGAGAG
	bis_seq_4_R	ACCCCCTCCCTAAAACTACAAC		bis_seq_3_R	CAAAAATAAACTTCACCTACCATAACA
	bis_seq_5_F	GTTGTAGTTTTAGGGAGGGGGT		bis_seq_4_F	TAAGAGGAAAAGTGATTTTTTAGTAATAGA
	bis_seq_5_R	CTACTTCTCCTCAACAACCAAAAAC		bis_seq_4_R	TTCATTTAAAAAACTACCTCAAACC
			SPRR2D	bis_seq_1_F	AGGTTATAAAGAATTGAGGAATTTAAGG
				bis_seq_1_R	AAAAAAATCATTATTCCCAATCAAA

Supplementary Table 3. qPCR primers

Target	Oligo name	Sequence (5' - 3')
HIC1	HIC1_F	CGACGACTACAAGAGCAGCA
	HIC1_R	TGCACACGTACAGGTTGTCA
RASSF1A	RASSF1A_F	ACCTCTGTGGCGACTTCATC
	RASSF1A_R	CGGTAGTGGCAGGTGAACTT
RASSF1C	RASSF1C_F	AGCTCGAGCAGTACTTCACC
	RASSF1C_R	TGCTGTTGATCTGGGCATTG
RASSF1 Total	RASSF1_F	TGCTGCGAAAGTTCTTGGTG
	RASSF1_R	CGCAAGTACACTTGGCCGT
PTEN	PTEN_F	ACCAGTGGCACTGTTGTTTC
	PTEN_R	TTAGCTGGCAGACCACAAAC
p14 (CDKN2A)	p14_F	CAGCCGCTTCCTAGAAGACC
	p14_R	ACGGGTCGGGTGAGAGTG
p16 (CDKN2A)	p16_F	CAACGCACCGAATAGTTACGG
	p16_R	ACGGGTCGGGTGAGAGTG
p15 (CDKN2B)	p15_F	GGGGACTAGTGGAGAAGGTG
	p15_R	CTGCCCATCATCATGACCTG
hTERT	hTERT_F	ATGCGTCGCAAACTCTTTGG
	hTERT_R	ACATGCGTGAAACCTGTACG
GAPDH	GAPDH_F	GATTTGGTCGTATTGGGCGC
	GAPDH_R	TTCCCGTTCTCAGCCTTGAC
ACTB	ACTB_F	TCCTCCCTGGAGAAGAGCTA
	ACTB_R	CCAGACAGCACTGTGTTGGC
