# Supplementary material for: Hit-and-run epigenetic editing prevents senescence entry in primary breast cells from healthy donors
Source: Nat Commun. 2017 Nov 13;8:1450. doi: 10.1038/s41467-017-01078-2 (PMC5684409; doi:10.1038/s41467-017-01078-2)
Supplement: Supplementary file 3 — Description of Additional Supplementary Files [file 41467_2017_1078_MOESM3_ESM.pdf]

Description of Additional Supplementary Files

File Name: Supplementary Movie 1
Description: **Seeding cells at high density prevents proliferation.** Primary myoepithelial cells from donor 1 were transfected with gRNAs (targeting *HIC1*, *RASSF1*, *PTEN* and *CDKN2A)*, dCas9 3A3L or 3A3LΔ and pMACs. Cells were magnetically sorted at 2 days post-transfection and at day 10 were reseeded into a 96 well plate at 3120 cells cm^{-1} (± 10%; high density). One image $hour^{-1}$ was taken and IncuCyte ZOOM software was used to convert the images into a video.

File Name: Supplementary Movie 2
Description: **Seeding cells at low density enables proliferation.** Primary myoepithelial cells from donor 1 were transfected with gRNAs (targeting *HIC1*, *RASSF1*, *PTEN* and *CDKN2A)*, dCas9 3A3L or 3A3LΔ and pMACs. Cells were magnetically sorted at 2 days post-transfection and at day 10 were reseeded into a 96 well plate at 780 cells cm^{-1} (± 10%; low density). One image $hour^{-1}$ was taken and IncuCyte ZOOM software was used to convert the images into a video.

File Name: Supplementary Data 1
Description: EPIC array data showing significantly >20% hypermethylated probes in 3A3L targeted vs. 3A3LΔ.

File Name: Supplementary Data 2
Description: Gene ontology analysis of differentially expressed transcripts in early passage, 3A3L targeted and 3A3LΔ targeted cells, 10 days post-transfection.

File Name: Supplementary Data 3
Description: Transcripts differentially expressed between dCas9 3A3L and 3A3LΔ targeted primary myoepithelial cells, 10 days post-transfection, after targeting CDKN2A, RASSF1, HIC1 and PTEN.
